# Secondary Structure Bead-Encoded Amphiphilicity Biases Peptide Self-Assembly Prediction in MARTINI Coarse-Grained Simulations

**DOI:** 10.1021/acsami.5c14754

**Published:** 2026-02-19

**Authors:** Marko Babić, Goran Mauša, Ivan R. Sasselli, Daniela Kalafatovic

**Affiliations:** † 37624 University of Rijeka, Faculty of Engineering, Rijeka 51000, Croatia; ‡ University of Rijeka, Center for Advanced Computing and Modelling, Rijeka 51000, Croatia; § 202635 Centro de Física de Materiales (CFM-MPC), CSIC-UPV/EHU, Donostia-San Sebastián 20018, Spain

**Keywords:** peptide self-assembly, MARTINI coarse-grained model, amphiphilicity bias, coarse-grained molecular dynamics

## Abstract

Sequence-dependent self-assembly of peptides yields ordered supramolecular structures with diverse nanotechnological applications. In the absence of simple design rules linking sequence to supramolecular morphology, coarse-grained molecular dynamics (CG-MD) simulations have become valuable tools for guiding the design of self-assembling peptides. The MARTINI model, despite the lack of explicit hydrogen bonding, can predict self-assembling sequences and structural features by introducing secondary structure-specific beads that adjust backbone polarity. Extended β-sheet encoding is typically used as input for short peptides, based on experimental observations. However, this assumption becomes increasingly unreliable beyond six to ten residues, where folded conformations begin to emerge. In this study, we investigated the effect of different secondary structure encodings on self-assembly simulations of hexapeptides and decapeptides using MARTINI 2.2p. The results confirmed that changes in the secondary structure encoding significantly impact the predicted self-assembly behavior, with AP scores for the same peptide varying by up to one unit—shifting from fully dissolved (AP ≈ 1) to well-aggregated states (AP > 2) in specific cases. This effect arises from alterations in overall peptide amphiphilicity caused by shifts in backbone polarity. However, the magnitude and direction of this influence depend on side-chain polarity and peptide length, making the resulting bias highly sequence-specific and difficult to anticipate or correct systematically. These findings emphasize the need to reevaluate the conventional use of extended β-sheet encoding (E-flag) and advocate for more native-like backbone representations in peptide self-assembly simulations.

## Graphical abstract

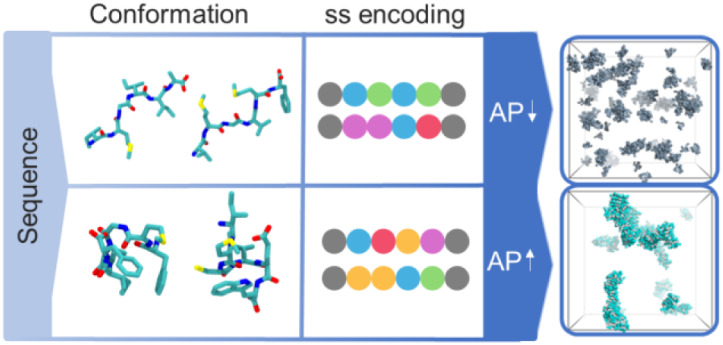

## Introduction

Peptide self-assembly is increasingly harnessed in nanotechnology and biomedicine to develop novel biomaterials for applications ranging from drug delivery and regenerative medicine to bioactive scaffolds, biosensors, catalytic systems and antimicrobial agents.^1–11^ The ability of peptides to spontaneously form highly ordered supramolecular structures through a sequence-dependent self-assembly process, driven by noncovalent interactions, further enhances their appeal for functional material design.^3^ The strong sequence dependence enables even short peptides to spontaneously organize into a wide range of nanostructures, including fibers, tubes, micelles, ribbons, and sheets.^12–17^ This diversity in morphology translates into a wide range of applications. For example, nanobelts can be engineered for thermal biosensing due to their stable and consistent morphological changes upon heating,^18^ nanofibers find applications in tissue engineering,^19^ spherical nanocarriers in drug delivery^20–22^ while hydrogels can be used as drug depots for stimuli-responsive release.^23^ The formation and properties of these nanostructures can be precisely controlled by solvent conditions,^24^ terminal capping and other covalent modifications^25,26^ as well as by the type and disposition of amino acids within the sequence.^4,16,27,28^ Given the extensive range of applications and the vast combinatorial space, encompassing approximately 10.24 trillion possible sequences using only 10 amino acids, there is a growing interest in accelerating the discovery of self-assembling peptides.

Computational tools have become instrumental in studying peptide self-assembly, leading to a deeper understanding of these systems at the molecular level and shedding light on their sequence-dependent behavior. Molecular dynamics (MD) simulations employ classical potentials and parameters, collectively known as force fields, that are valuable for capturing the behavior of these systems at relevant scales, enabling comparison across sequences and providing detailed insights into the role of specific interactions in the resulting structures. Despite advances made with all-atom (AA) simulations, the use of coarse-grained (CG) models such as MARTINI, simplify molecules, reducing the number of simulated particles and the associated computational load, considerably extending the accessible size and time scales. This makes CG models ideal for studying larger assemblies and longer self-assembly processes with computational cost reduced by a few orders of magnitude.^29,30^

MARTINI is one of the most widely used CG force fields, valued for its efficiency and accuracy in modeling large biomolecular systems.^29,31^ This force field reduces the number of interaction sites by grouping atoms into “beads” at a 4:1 ratio, with smaller (3:1) and tiny (2:1) beads also available in the latest version.^32^ These larger particles permit longer time steps (20 to 40 fs) compared to AA simulations (1 to 2 fs) and in combination with smoother energy landscapes allow for substantially longer simulation times of large systems.^30,33^ Due to these advancements, the MARTINI force field has proven to be a valuable tool for studying peptide aggregation and self-assembly processes, supporting the development and optimization of new peptide-based materials.^29,34–37^ It has successfully reproduced the formation of micelle, fiber, and tube nanostructures previously observed only experimentally^38,39^ including studies under constant pH,^40^ different concentrations,^41^ combinations of different peptides in coassemblies,^42–46^ and intermolecular mobility.^47,48^

The MARTINI model stands out for its suitability in high-throughput approaches, enabling efficient screening of large peptide libraries and rapid exploration of the sequence space, such as exhaustive screening of all 400 dipeptide^49^ and 8000 tripeptide permutations.^50^ In these studies, Frederix et al., correlated a measure of reduction in the total solvent accessible surface area (SASA) between the starting and final measurements with experimental evidence of self-assembly confirmed by TEM, AFM, and FTIR. This ratio of the initial and final SASA was named aggregation propensity (AP) score of the peptide and, if its value exceeded two, the experimental data showed self-assembly. Recent studies have been extended to peptide amphiphiles^51^ and to the search for liquid–liquid phase separation, combining AP with cluster analysis.^52^ However, despite the computational acceleration that MARTINI provides, the search space increases exponentially with peptide length (20^*n*^ possible sequences for peptides of length *n*), making exhaustive sequence screening impractical for longer peptides. To overcome some of these limitations, CG-MD simulations have been combined with machine learning (ML) to provide a smart screening platform and accelerate the discovery of new candidates.^53,54^ Furthermore, generative models able to suggest new self-assembling sequences from unexplored regions of the peptide space were complemented with CG-MD to validate the new knowledge and aid in the selection of candidates for experimental validation.^55^ While these hybrid approaches show great promise, their success ultimately depends on the accuracy and reliability of the CG models used, underscoring the need to critically assess the validity of these methods and their agreement with experimental observations.

Originally developed for lipid membranes, MARTINI has since been extended to proteins and peptides.^56^ MARTINI 2.1 protein version was modified to reduce the excessive hydrophobicity of certain side chains, leading to version 2.2.^57^ The overestimated hydrophobicity present in the MARTINI models was recently refined in MARTINI 3, leading to improved protein dynamics and more accurate modeling of protein–ligand binding. However, the excellent performance of version 2.1 for short peptides, particularly dipeptides, was compromised in version 2.2, and further exacerbated in MARTINI 3 by reducing the force field’s ability to model short-peptide self-assembly.^34,35,44^ However, recent reports indicate that for peptides longer than 10 amino acids, MARTINI 3 may successfully reproduce experimentally validated self-assembly behavior.^58^ In contrast, for shorter peptides, improvements to the force field are still required to ensure accurate modeling, similarly to the case of intrinsically disordered proteins, where specific reparameterization was necessary to capture their phase transitions. Thus, version 2.1 appears optimal for dipeptides and version 2.2 for longer peptides up to 10 residues, further enhanced by the use of polarizable beads that improve the treatment of electrostatic interactions.^35^

The resolution loss in the MARTINI model prevents it from explicitly reproducing hydrogen bonding, which is critical for secondary structure formation in peptides. To compensate for this limitation, the secondary structure (ss) information must be explicitly provided as input. In addition to adjusting the bonded terms, this input modifies the polarity of the backbone beads, which can be critical to modulate peptide aggregation. Recent studies analyzing the impact of the type and arrangement of beads on aggregation have revealed inconsistencies between coil and β-sheet DSSP encodings in tetrapeptides. Specifically, nonpolar backbones showed higher AP scores and distinct morphologies, as indicated by moment of inertia ratios.^59^ The influence of backbone encoding on AP, combined with the ability of hexapeptides and longer sequences to adopt folded structures not observed in shorter peptides,^60,61^ raises concerns that default use of the E-flag may introduce bias in sequence screening approaches. The latest MARTINI 3 model removes this dependence by employing a consistent backbone polarity across all ss encodings. However, its optimization to reproduce protein dynamics has reduced its ability to replicate the success of previous versions in simulating short peptides. One of the key issues is an overall increase in hydrophilicity, enhanced by the stronger influence of charged termini and side-chain–side-chain interactions, which outweigh side-chain–water interactions.^34,35,57^

To date, the specific role of backbone beads in modulating peptide aggregation and self-assembly remains underexplored. In this paper, we systematically assessed the effect of backbone bead types on the aggregation behavior of hexa- and decapeptides within the context of the MARTINI 2.2p model. First, we examined intrinsic backbone interactions employing a model system using only glycines (Gly), which have no side chain in MARTINI. Next, we evaluated homopeptides featuring side chains of varying chemical properties—phenylalanine (Phe, aromatic), isoleucine (Ile, aliphatic), methionine (Met, nonpolar), asparagine (Asn, polar), aspartic acid (Asp, negatively charged), and lysine (Lys, positively charged)—to assess their modulatory effects on backbone behavior. Finally, we applied ss encodings based on predicted near-native conformations to determine whether nonpolar backbones introduce aggregation propensity biases, and whether such effects persist across different peptide lengths (Figure 1). Custom input monomer conformations were derived from cluster analysis, yielding more relaxed and polar structures, or from PEPFOLD3 predictions, which result in highly ordered and more hydrophobic conformations. This study aims to determine whether the incorporation of near-native conformations of monomers can offer a viable alternative to conventional E-flag encoding. This could lead to an improved predictive accuracy of CG-MD simulations, especially when modeling initial self-assembly stages, and make them comparable to experimental validations.

**Figure 1:**
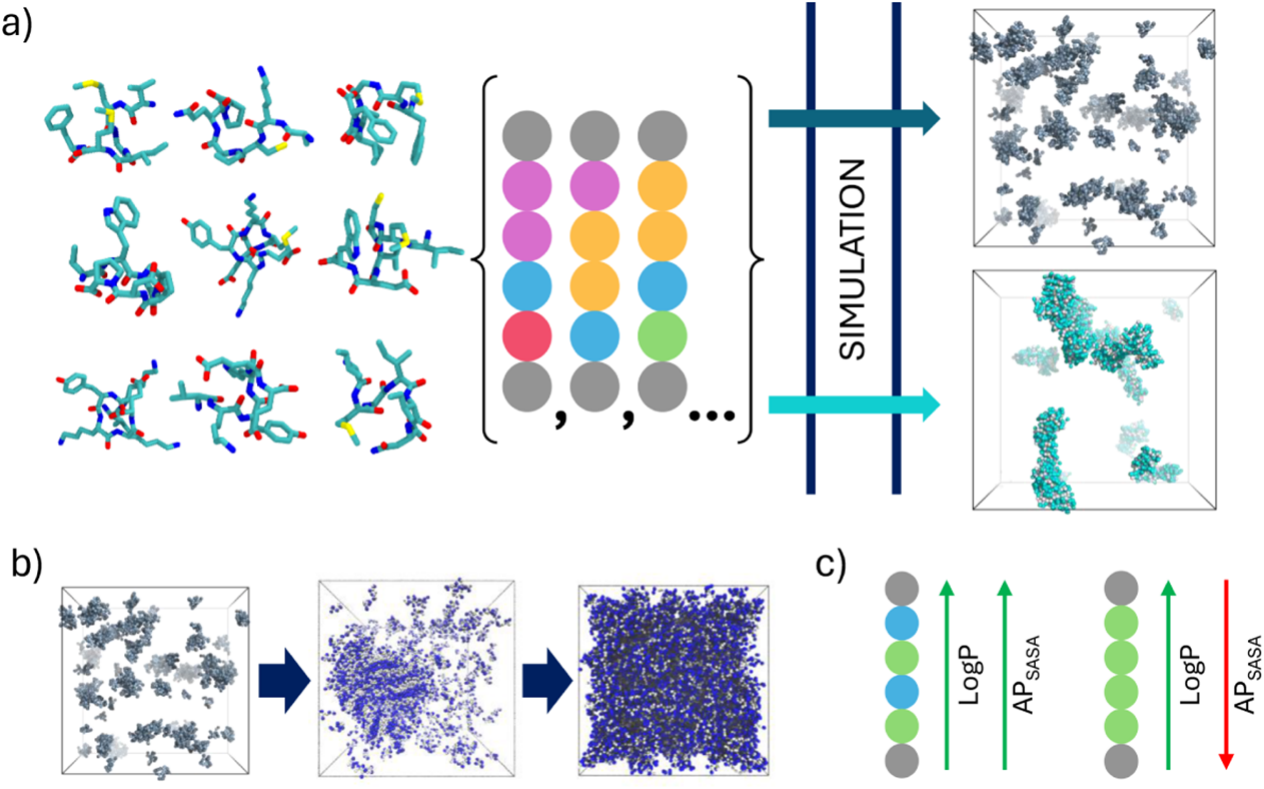
Roadmap for assessing the influence of secondary structure input on peptide self-assembly in MARTINI. To determine whether commonly used backbones introduce aggregation propensity bias a heterogeneous set of hexapeptides and decapeptides with near-native backbone encoding was simulated along polar (C-flag) and nonpolar (E-flag) backbone variants to evaluate the impact of (a) different encodings and (b) peptide concentrationon aggregation, through assessment of AP scores, and (c) AP to log*P* correlations.

## Methods

### Building the PDB Files

PDB files were built for 34 peptides used in this study. The model systems were based on 14 homopeptides: *Gly*_6_, *Gly*_10_, *Phe*_6_, *Phe*_10_, *Ile*_6_, *Ile*_10_, *Met*_6_, *Met*_10_, *Asn*_6_, *Asn*_10_, *Asp*_6_, *Asp*_10_, *Lys*_6_, *Lys*_10_. The real-world scenario was based on 20 AI-generated heteropeptides from previous work:^55^ AKCPQP, FMGIIF, IMCIEW, IMGIIA, IMGIIN, MKYKEE, VKYKEE, VKYKKE, VMGIMF, VWPPDP, FATAAGGNMF, FATAAGGNNF, FGDAAGGNNF, FGDAAGGNTF, FGDAAGGNTT, VNGYSPKWPG, VRMHHKKNQG, VRMHHPKWPG, VRMHHRKEQG, VRMHHRKNQG. Peptide PDB files were built (i) using PyMOL (v2.5.2)^62^ without additional modifications, or (ii) built with PyMOL and run through a cluster analysis or (iii) using PEPFOLD3 ^63^ and downloaded from the website in PDB format.

### Determining Peptide Conformations

The ss encoding for the CG simulations was derived from two sources: (i) the most probable folded conformation predicted by PEPFOLD3, and (ii) an ensemble of structures obtained from cluster analysis of AA-MD simulations in solution. To obtain the PEPFOLD3 input, FASTA format of the peptides sequences was written into the PEPFOLD3 algorithm that provided a set of possible folds. “Model 1” as the most likely fold of each peptide was chosen as representative conformation, based on the algorithm’s instructions.^63,64^ Cluster analysis was performed on conformations obtained from a 10 ns atomistic simulation of a single peptide in water with the CHARMM36m force field. The atomistic simulations used steepest descent minimization with 5000 step limit, a 125 ps NVT equilibration with a 1 fs time step, which used Nosé–Hoover thermostat, and NPT production run using Nosé–Hoover thermostat and Parrinello–Rahman barostat with a 2 fs time step. The system neutralized the charge of the peptides by adding a minimal number of K or Cl ions, ranging from 0 to 10 depending on the peptide charges, and the box was set to be 10 nm from the edges of the largest dimension of the tested peptide through the CHARMM-GUI website. Clustering was performed using the GROMOS algorithm with a 0.3 nm backbone RMSD cutoff for hexapeptides and 0.4 nm backbone cutoff for decapeptides. This variation in cutoff was made because the decapeptides formed too many clusters using the 0.3 nm cutoff to determine a dominant structure. The representative of the most populated cluster was used as the structure for further simulations representing the most common conformation of the peptide in solution.

### Extracting and Employing DSSP Codes for AA to CG Conversion

Both cluster analysis and PEPFOLD3 fold predictions were analyzed in GROMACS to extract the DSSP flags. DSSP command in GROMACS was run with an option for predicting absent hydrogen atoms (−hmode dssp) and a neighbor search method (−nb yes), which might bias toward detecting secondary structures with a higher degree of hydrogen bonding. The conversion process from AA to CG files was carried out using the martinize.py script selecting the MARTINI 2.2p CG force field with the following ss flags: (i) extracted PEPFOLD3 fold predictions and cluster analysis DSSP flags (Table S6, Figures S6 and S7), (ii) the standard E-flag that represents the extended β-sheet conformation, (iii) C-flag (the ″∼″ tilde symbol or no ″-ss″ command was used) to encode “coil” DSSP that represents unstructured regions, or (iv) combinations of E and ∼ flags to implement maximally nonpolar or polar bead configurations in the backbone. For MARTINI 3 CG simulations, the “vermouth” program was used to convert AA to CG representations using Cluster, E-flags, or PEPFOLD3 flags. C-flag parameters were identical to Cluster in MARTINI 3 for the group of peptides included in this study.

### CG Simulation Setup

For all 34 peptides, of which 14 homopeptides and 20 heteropeptides, simulations were performed from randomly distributed peptides in solution using polarizable water. Different concentrations were used by inserting the number of peptides equal to a total amino acid count of 1200, 4800, or 14400 per 20 × 20 × 20 nm box. The corresponding concentrations for the hexapeptides were 42, 166, and 498 mM, while those for the decapeptides were 25, and 100 mM. For decapeptides, simulations with 14400 amino acids were not performed because there were no increases in AP from 25 mM (Figure S8a, cluster encoded) to 100 mM simulations (Table S9).

Each simulation underwent a three-step energy minimization, a two-step equilibration, and a dynamics run lasting 1000 ns with a 20 fs time step (or 4000 ns of “effective time”). The minimization and equilibration employed 4000 kJ/mol backbone constraints. The initial peptide setup was minimized prior to the addition of water. After adding PW, a “soft-core” minimization lasting 20,000 steps of 20 fs was performed. The system was then minimized again using standard steepest descent algorithms for 50,000 steps with shorter 10 fs time steps. Equilibration was conducted in two phases: an initial short V-rescale thermostat and Berendsen barostat isotropic equilibration, followed by a more extended Nosé–Hoover, thermostat and Parrinello–Rahman barostat in a semi-isotropic system.^55^ Simulations using 14400 amino acids per box (2400 peptides) had problems performing the second equilibration used for the rest of the simulations due to the instability of nonstochastic algorithms in such crowded molecular systems. For this reason, these simulations followed the same protocol until minimization and then were run directly in a production run with a V-rescale thermostat and Berendsen barostat for 1000 ns with a 20 fs time step. In addition, we simulated five selected hexapeptides at three varying concentrations (200, 800, and 2400 peptides per box) using MARTINI 3. These systems were successfully minimized with a single steepest descent step and then directly run for 1000 ns production with a V-rescale thermostat and a Berendsen barostat. The total wall time for each simulation was approximately 48 h on 10 Intel Xeon E5-2690v3 processors, while it required approximately 48 h on 1 node with the 48-core Intel Xeon Platinum 8168.

### Aggregation Propensity Analysis

The analysis was performed by visual inspection through VMD software, and an analysis of AP_SASA_ using the GROMACS SASA tool.^65^ An AP_SASA_ score is the solvent accessible surface area (SASA) ratio between the initial and average SASA of the last 5% of the simulation, after assessing the equilibration of aggregate formation with SASA plots. The ratios were averaged and their standard deviation was calculated and plotted. Water contacts were used to determine the molecular disposition in the aggregates and were performed by counting CG waters in the first sphere of hydration using a cutoff radius of 0.7 nm for each backbone bead of the peptide. The water contacts were multiplied by four due to a single CG water representing 4 water molecules.

### Statistical Analysis

The statistical significance of the difference between the C-flag simulations and the backbone encoded simulations, for the 20 heteropeptides, were calculated using paired repeated measures *t* test. The difference among E-flag, Cluster, and PEPFOLD3 encoding was statistically analyzed with repeated measures analysis of variance (RM-ANOVA) and posthoc analysis for statistically significant differences in the AP scores with a Greenhouse–Geisser correction to account for the lack of sphericity of the data. The multiple pairwise hypothesis tests used the Bonferroni correction to reduce Type I (false positive) errors.

### Log*P* Correlation

The logarithm of the octanol–water partition coefficient (log*P*) of the PDB files created in PyMOL was calculated using the VEGA online tool.^66^ The correlation between the calculated log*P* values and the AP scores was assessed through the coefficient of determination (*R* ^2^).

## Results and Discussion

The MARTINI model uses bead-specific encodings to modulate backbone polarity and bonded parameters, thereby enforcing secondary structure formation to compensate for the absence of explicit hydrogen bonding. The ss encoding in MARTINI employs the Dictionary of Secondary Structure of Proteins (DSSP) classification.^67^ Although ss assignments modify bonded terms between backbone beads, these changes are relatively modest and do not rigidly constrain conformational flexibility, often requiring additional restraints to preserve folded structures.^68,69^ Thus, the primary effect of the ss encoding in MARTINI is the modulation of backbone bead polarity based on the hydrogen bonding pattern typical of each ss conformation (Figure 2a). DSSP classifications assign residues to nine structural types, but in MARTINI, these are reduced to two backbone bead categories based on hydrogen bonding trends: polar (P) beads are used for unstructured (coils, bends, turns, π-, polyproline, and 3_10_-helices, and isolated β-bridges) while nonpolar (N) beads are assigned to α-helix, β-sheet, and hydrogen-bonded turns, whose internal hydrogen bonds reduce backbone polarity.^56^ Note that multiple consecutive “H” DSSP codes for longer α-helix chains can create a special condition that encodes Na and Nd beads (or N0 beads for Ala, Pro, and Hyp) but were omitted as not relevant to this study.

**Figure 2:**
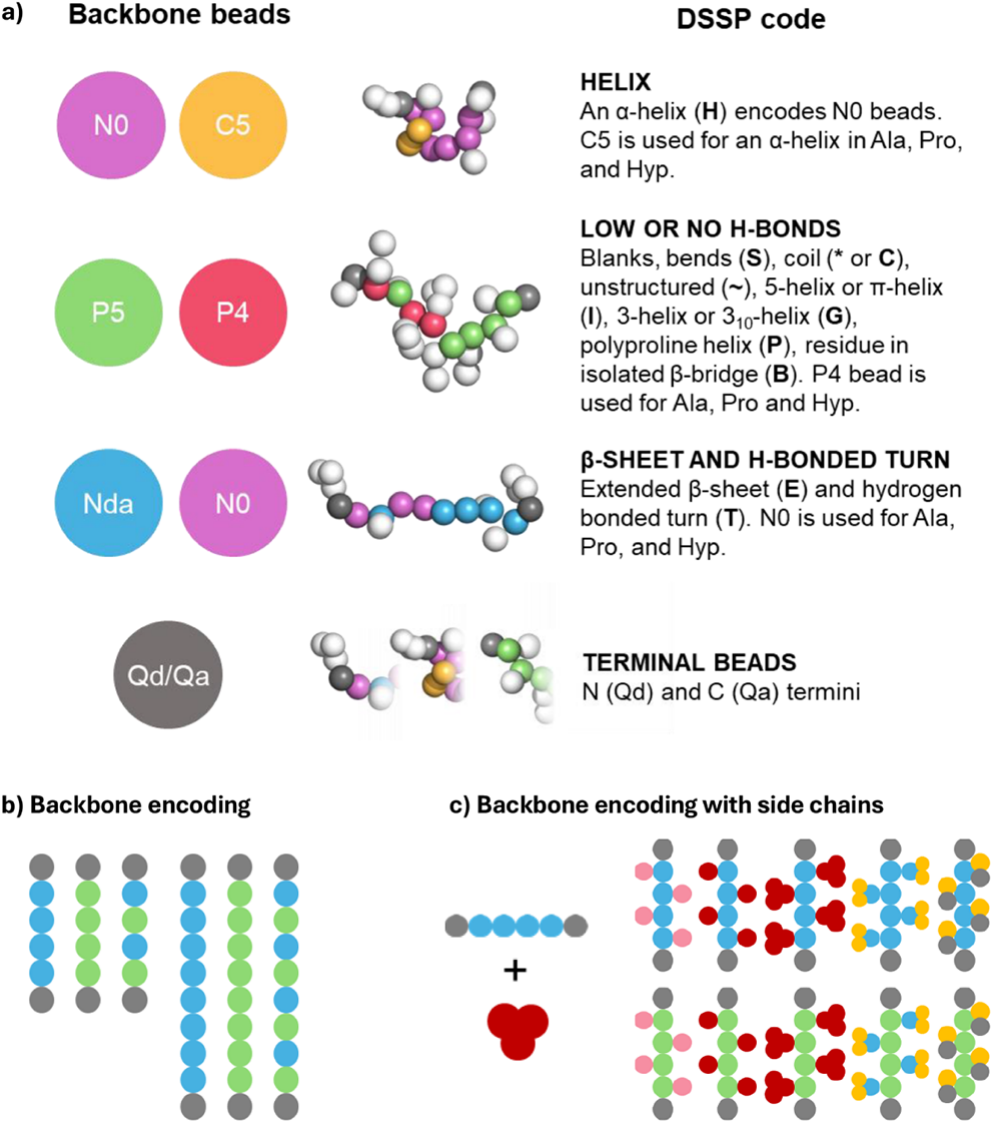
Beads and peptide encoding overview. (a) Backbone beads used in MARTINI 2.2p and the corresponding DSSP codes. State-of-the-art CG-MD simulations for obtaining the AP_SASA_ scores are performed using nonpolar (Nda) beads that correspond to the extended β-sheet DSSP code “E” for each position of the sequence except the termini residues. The specific role of backbone beads on the aggregation behavior of hexa- and decapeptides was assessed using (b) polyglycines with different polarities and (c) homopeptides with added side chains with specific chemical properties.

For peptide self-assembly simulations, the extended β-sheet DSSP flag (E-flag) is most commonly used, introducing nonpolar backbone beads expected to promote β-sheet-like organization in line with experimental observations (Table S1). However, longer peptide sequences can exhibit more heterogeneous conformations, for which the application of the E-flag may introduce a structural bias that leads to inaccurate aggregation propensity (AP) estimations. To isolate and understand how backbone polarity and its distribution influence the aggregation, we first focused on simplified model systems, initially composed solely of glycine residues (Figure 2b), and then extended to homopeptides featuring side chains of varying chemical nature (Figure 2c). Subsequently, to assess a real-world scenario, more complex sequences consisting of hexapeptides and decapeptides, previously obtained by generative AI,^55^ having different amino acid composition and varying aggregation range, were simulated using a near-native backbone encoding to account for their possible conformational preferences and compared to the E-flag and C-flag encodings.

### Analyzing Backbone-Only Interactions with *Gly*_6_ and *Gly*_10_

Glycines in the MARTINI force field are represented as a single backbone bead. Therefore, they offer a unique, simplistic model to analyze how backbone bead interactions for polar (P) and nonpolar (N) beads affect aggregation without the interference of side chains. For this purpose, we built a hexa- and a decapeptide containing only glycines (*Gly*_6_ and *Gly*_10_) and then encoded their backbones using different ss patterns. We encoded Gly homopeptides with all N or all P beads, followed by various N–P bead patterns to observe how AP scores, water contacts, and morphologies change. Considering that N- and C-termini must be set to charged beads, Qa and Qd, respectively, hexapeptides have 4 residues to set (residues 2 to 5) and decapeptides 8 (2 to 9). Therefore, we considered six options for the hexapeptide: NNNN, NNPP, NPNP, NPPN, PNNP, and PPPP; and seven for the decapeptide: NNNNNNNN, NNNNPPPP, NNPPPPNN, NPNPNPNP, PPNNNNPP, PPNNPPNN, and PPPPPPPP. It is important to note that these combinations consider fully polar (PPPP and PPPPPPPP), fully nonpolar (NNNN and NNNNNNNN), amphiphiles (NNPP and NNNNPPPP), and more complex bead combinations.

Peptide simulations in the literature predominantly use 13 × 13 × 13 nm water boxes containing hundreds of peptide copies, at concentrations ranging from 30 to 400 mM for durations between 12.5 and 1200 ns (Table S1). In our study, each system was simulated for 1000 ns to ensure adequate sampling of aggregation behavior and allow comparison across peptide lengths and concentrations. A similar setup was previously reported for 300 dipeptides^49^ and tripeptides^50^ in the box, which corresponds to 46 and 69 amino acids per nanometer of the box side, respectively. To maintain a comparable packing density for longer peptides (in our case, hexa- and decapeptides) while also accounting for the minimum number of peptides required and the improved structural formation observed in larger systems, we increased the box size to 20 × 20 × 20 nm. Based on an average of ≈60 amino acids per nanometer, we set 1200 amino acids as the minimum system size, corresponding to concentrations of 42 mM for hexapeptides and 25 mM for decapeptides. We also tested higher concentrations by simulating systems with 4800 amino acids (166 mM for hexapeptides, 100 mM for decapeptides), and 14,400 amino acids (498 mM) for hexapeptides only. The highest concentration was not used for decapeptides, as no additional aggregation was observed at 100 mM. By simulating systems across a range of concentrations, yet within values commonly reported in the literature, we aimed to assess potential concentration effects on AP scoring, as well as morphological variations arising from changes in molecular crowding.

Visualization of the final simulation frames revealed the formation of elongated aggregates for both hexa- and decapeptides with all bead combinations (Figures 3, S1 and S2), consistent with expected peptide self-assembly behavior. The corresponding AP values, after equilibration of the simulations (Figure S3), support this, with all systems exhibiting values greater than 1.5. While some decapeptides display the highest AP values overall, they do not consistently outperform hexapeptides, indicating that bead distribution plays a more critical role than peptide length. No clear concentration dependence was observed across these simulations. Interestingly, the lowest AP values were observed for fully nonpolar chains (≈1.6 for NNNN and NNNNNNNN), while most other sequences showed similar values: 1.97 to 2.11 for hexapeptides and 2.00 to 2.21 for decapeptides. Only the decapeptide with two perfectly separated regions (NNNNPPPP) yielded intermediate AP values (1.86). In particular, alternating sequences (NPNP and NPNPNPNP) yielded higher AP values than sequences with clustered beads (NNPP, NPPN, PNNP, for hexapeptides and NNPPPPNN, PPNNNNPP, and PPNNPPNN, for decapeptides). This result challenges previous assumptions that nonpolar particles promoted aggregation, as fully polar chains (PPPP and PPPPPPPP) exhibited AP values ≈0.4 to 0.5 units higher than their fully nonpolar counterparts (Figure 3 and Table S2). This observation can be rationalized by the strong self-interactions of polar beads in the Martini force field, which outweigh their favorable solvation and ultimately drive aggregation.

**Figure 3:**
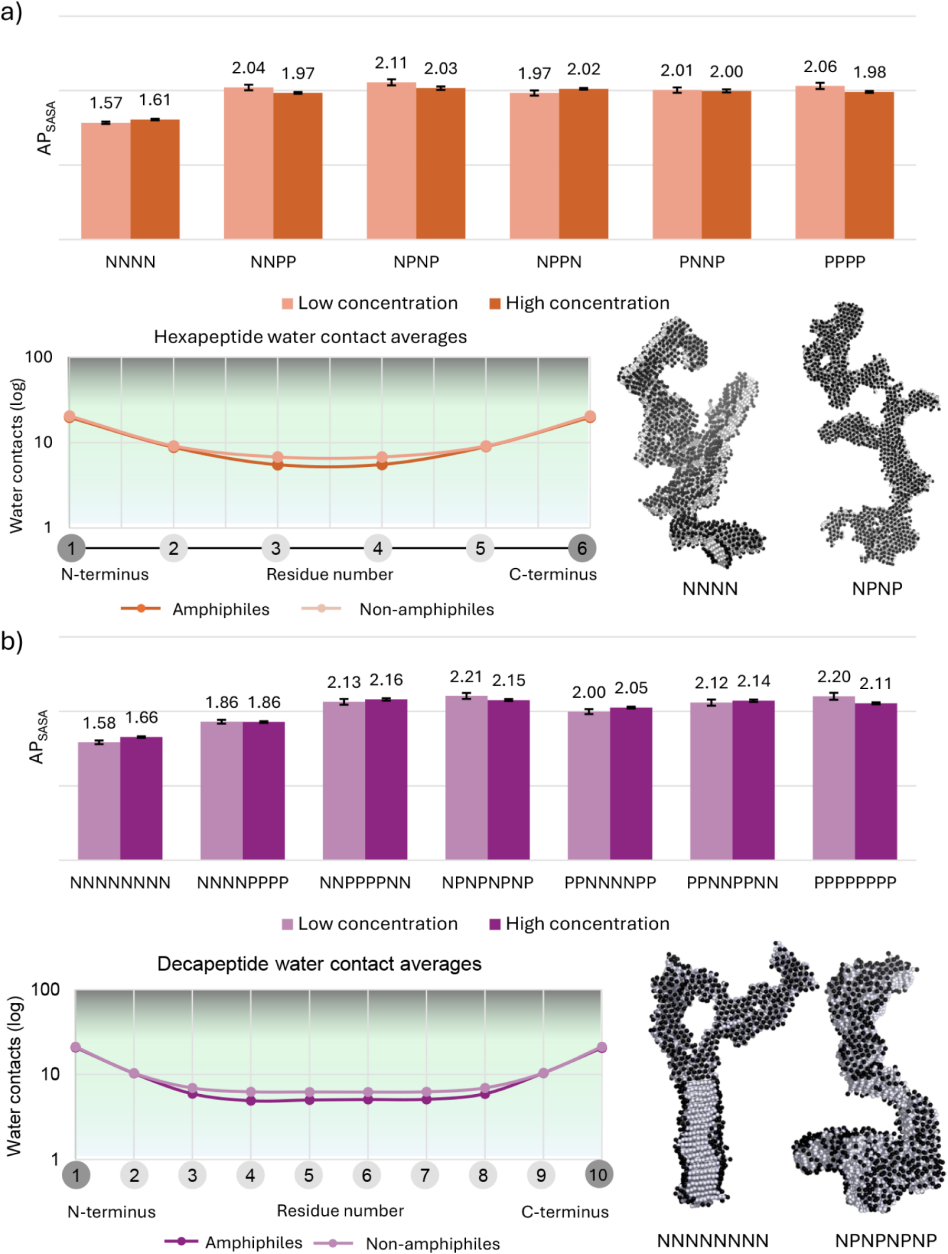
Glycine homopeptides. AP scores, water contact mapping, and images of (a) glycine hexa- and (b) decapeptides with backbone encoding based on various combinations of P and N beads. The images show examples of morphologies of the nonpolar (NNNN; NNNNNNNN) and amphiphilic (NPNP; NPNPNPNP) high-concentration snapshots of the final simulation frames. Low and high concentrations correspond to 42 mM and 166 mM for hexapeptides, and 25 mM and 100 mM for decapeptides, respectively. Water contact graphs detect contacts within a 0.7 nm radius and the contact count is represented on a base 10 log-scale.

Furthermore, visual inspection revealed a general stacking of peptides resembling a monolayer. However, despite the presence of small lateral areas and some branching (Figures 3, S1 and S2), the assemblies failed to show a clear trend toward 2-D growth. Instead, the growth is predominantly 1-D, more consistent with a β-sheet-like organization than with bilayer formation. In these β-sheets, P and N beads stack in the core, while the charged termini remain exposed to the solvent (Figures S1 and S2). This arrangement can be quantitatively analyzed through water contact graphs, which measure the exposure of each residue to solvent by averaging the number of water molecules in the first hydration shell of each backbone bead in the final assembly. These graphs show higher hydration levels for the first and last two residues in both hexa- and decapeptides, while the central residues consistently exhibit lower and more uniform hydration (Figure 3, Tables S3 and S4). This hydration profile aligns well with the β-sheet-like structures observed in a related study.^58^ The amphiphilic chains containing combinations of N and P beads (NNPP, NPNP, PNNP, NPPN, NNNNPPPP, NNPPPPNN, NPNPNPNP, PPNNNNPP, and PPNNPPNN) display lower overall hydration in the central residues (residues 3 and 4 for hexapeptides and 3 to 8 for decapeptides). In light of previous studies, the ability of peptides to exclude water from the core in such simulations has been shown to correlate with increased intermolecular order.^48,58^ Thus, these findings indicate that it is the distribution of N and P beads that promotes the formation of higher-order assemblies.

### Effect of Side Chains on Aggregation in Homopeptide Model Systems

After assessing the effect of the backbone bead polarity using Gly-only model systems, we expanded the study to include homopeptides based on amino acids with side chains having diverse physicochemical properties (Figure 4c), to evaluate how side chains modulate aggregation behavior. Ile was selected for its aliphatic side chain, represented in MARTINI by a single C1 bead, the most hydrophobic bead type, and was therefore expected to promote aggregation. Phe is also hydrophobic but aromatic; its side chain is modeled using three small hydrophobic beads (SC4) arranged in a triangular geometry to capture planarity and enable potential π–stacking interactions, which are also known to promote aggregation. Met, despite containing a sulfur atom, is considered nonpolar and is represented by a single C5 bead. Asn is polar; however, in the MARTINI model, its side chain is represented using a nonpolar Nda bead. Asp, a negatively charged amino acid, and Lys, a positively charged amino acid, were selected to evaluate the effect of charged side chains. Asp is modeled with a Qa bead that carries a single negative charge, while Lys is modeled with a C3 representing its long carbon chain connected to a Qd bead that carries a single positive charge. We evaluated how these different side chains influence aggregation through AP analysis at the lowest concentration tested (200 hexapeptides or 120 decapeptides per box).

**Figure 4:**
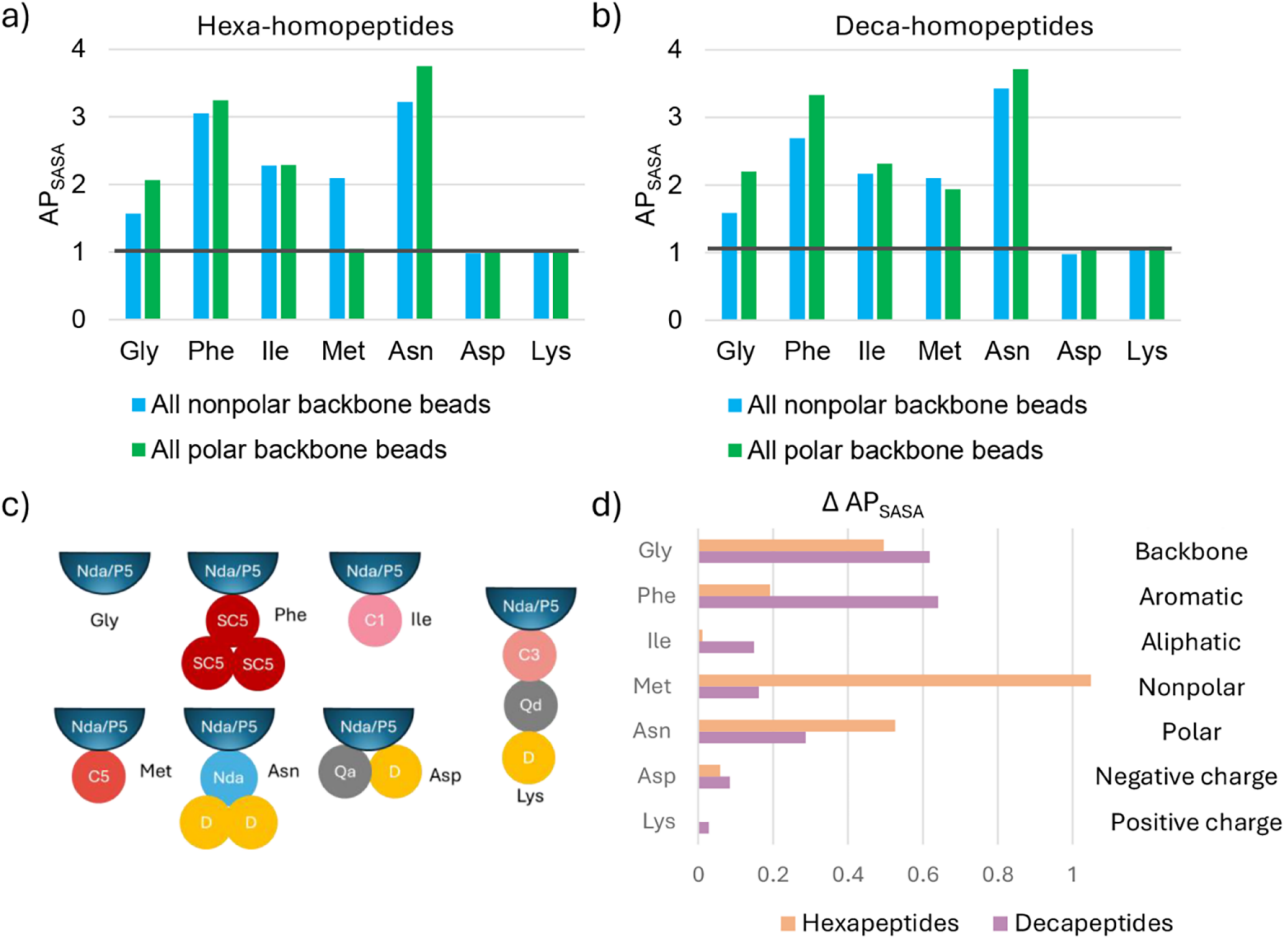
Homopeptides with fully polar or nonpolar backbones with different side chain properties. The histograms show AP scores in (a) hexa-homopeptides and (b) deca-homopeptides with aromatic (Phe), aliphatic (Ile), nonpolar (Met) or polar (Asn), negatively (Asp) or positively (Lys) charged side chains alongside Gly homopeptides to compare how backbone and side chain properties interact to influence AP_SASA_. (c) Schematic representation of side chain beads of each used amino acid. (d) The ΔAP_SASA_ when subtracting the AP scores of the homopeptide with polar and nonpolar backbones.

The results after equilibration (Figure S5) are presented in conjunction with those of the Gly homopeptides for comparison (Figures 4). At first glance, it is evident that the side chains modulate the aggregation behavior of the peptides (Figures 4a,b, S4, Table S5). Asp and Lys side chains inhibit aggregation in both peptide lengths and backbone polarities, showing AP values close to 1. In contrast, Phe and Asn enhance aggregation with either backbone polarity, exhibiting the highest AP scores among the amino acids tested. Ile shows enhanced aggregation only with nonpolar beads, compared to Gly. Interestingly, polar backbones increase aggregation in all cases except for Met, where aggregation is fully suppressed in hexapeptides. These results demonstrate that side chains modulate the influence of backbone bead polarity on the aggregation behavior.

To better visualize the changes in aggregation propensity induced by backbone polarity, the ΔAP metric is shown in Figure 4d. The data reveal that the polar backbones strongly enhance aggregation for Asn in hexapeptides, whereas for decapeptides, the effect is most pronounced in Phe. However, the maximum ΔAP values in both cases are comparable to those observed with Gly homopeptides, suggesting that the Gly system provides a useful baseline for assessing the maximum influence of the backbone polarity. Ile shows the smallest differences overall, with negligible ΔAP in hexapeptides. Met is a particularly interesting case, it not only shows the sole negative effect of increasing backbone polarity but also displays the strongest such effect among all residues tested. However, this effect is negligible in decapeptides. We conclude that side chains modulate the aggregation by enhancing the effect of polar backbone beads in diverse and nonintuitive ways. Because these effects already appear in homopeptides, composed of a single type of amino acid, the addition of sequence heterogeneity will likely amplify this unpredictability. Side chains clearly tune how backbone beads, especially polar ones, promote aggregation, but the direction and magnitude of this tuning are not straightforward to anticipate. Aggregation is reduced in the presence of charged side chains, whereas it is enhanced for most other residues that maintain greater AP values with polar backbone beads, particularly aromatic and polar side chains. Nevertheless, the presence of apolar side chains (C1 for Ile and C5 for Met) mitigates the increased aggregation associated with polar backbones, rendering differences negligible for *Ile*_6_ and reducing aggregation for *Met*_6_. This behavior likely arises because apolar side chain beads disrupt the strong backbone–backbone packing that otherwise promotes aggregation, leading even *Met*_6_ to remain dispersed in solution rather than forming aggregates. Increasing the peptide length seems to shift the balance toward backbone-dominated behavior, with polar backbones displaying higher AP scores for *Ile*_6_ and reduced differences for *Met*_6_. Lastly, while most of these differences do not correspond to substantial changes in aggregate morphology, except in the case of *Met*_6_ (Figure S4a,c), the observed AP variations of up to 0.4 to 0.6 could affect the selection of peptides in high-throughput screening protocols.

### Encoding Secondary Structure Information in the Backbone

Building on the observed influence of side chain chemistry on how backbone bead polarity modulates aggregation, we next examined how more complex ss encoding affects AP scores. This is critical since AP is frequently used as a screening metric. Any encoding-related bias could affect the selection of candidate sequences in high-throughput workflows. Unlike in proteins, the ss of peptides cannot be easily derived from experimental structures because of their intrinsic flexibility and lack of stable tertiary folds. To address this, we used two complementary approaches to assign ss of monomeric states: (i) the most probable folded conformation predicted by PEPFOLD3, and (ii) the dominant structural states obtained from the cluster analysis of AA-MD simulations in solution. Given that the peptides analyzed in this study are 6 or 10 residues long, PEPFOLD was chosen as specifically developed and benchmarked for de novo modeling of peptides, typically from 5 to 15 amino acids,^63^ providing an efficient approach for exploring their conformations. Cluster-derived conformations represent the most frequently sampled backbone configurations under realistic solvent conditions, while PEPFOLD3 outputs correspond to idealized low-energy states that peptides may adopt intermittently. As a result, we defined two distinct sets of ss labels, referred to hereafter as Cluster and PEPFOLD3, to investigate how more native-like backbone encodings influence aggregation behavior in MARTINI simulations. In addition, fully extended chains (all E-flags) and fully unstructured chains (all C-flags) were included to cover the full range of polarity, from nonpolar (E-flag) to polar (C-flag) to systematically assess the effect of this parameter. This study aims to determine whether the incorporation of near-native monomer conformations can offer a viable alternative to conventional E-flag encoding and to systematically evaluate the overall effect of secondary-structure polarity on self-assembly predictions.

The output structures from the Cluster and PEPFOLD3 approaches show notable differences, with the latter producing more folded conformations than the former (Figures S6 and S7). The analysis of ss labels (Table S6) supports this observation: Cluster-derived structures contain a higher proportion of undefined regions (^∼^), while PEPFOLD3-predicted structures exhibit more well-defined secondary structure elements. Following unstructured regions, polyproline (P) helices are the most prevalent ss assignment in the Cluster-derived labels. However, it is important to note that in MARTINI, both unstructured (^∼^) and P are treated equivalently to coil (C), and therefore give rise to highly polar backbone chains. The bend (S) flag appears in 9 sequences, whereas the turn (T) flag is present in only one. Since only the T flag introduces nonpolar backbone beads, we can conclude that, across the Cluster-derived labels, all but two backbone beads are polar. In contrast, PEPFOLD3 outputs include a higher number of α-helical (H) and turn (T) assignments, which reduce chain polarity through the incorporation of nonpolar backbone beads. Several 3_10_ helices (G) are also present, which are modeled with polar beads in MARTINI, unlike α-helices. Additional assignments include some unstructured (^∼^), bend (S), and extended (E) structures. Importantly, the presence of E-flags associated with β-sheet conformations is minimal for the monomeric states, contrasting with the common assumption that β-sheet-like structures dominate in the final stages of peptide self-assembly. Still, some sequences are composed exclusively of H, T, and E elements, resulting in predominantly nonpolar backbones, while a few amphiphilic examples combine these nonpolar regions with segments assigned to polar categories (^∼^, P, S, G), leading to mixed polarity when translated into MARTINI bead types. Therefore, comparing the four encoding sets, the ranking as a function of the backbone polarity would be *C*-*flag* > *Cluster* > *PEPFOLD*3 > *E*-*flag*. These four sets were simulated at the lowest concentration tested, with 1200 amino acids, giving 200 hexapeptides and 120 decapeptides and concentrations of 42 and 25 mM, respectively. Convergence was achieved in all cases within the simulated time window, allowing for the characterization of equilibrated self-assembled states (Figures S13 and S14).

The variability in the results across different ss encodings is evident (Figures 5, S8a, S9, S10, S11, S12) and no single flag consistently yields the highest AP for all cases. However, the lowest AP values for 18 of 20 peptides had C-flag encoding. Only PEPFOLD3 labels resulted in lower AP scores for two peptides, both of which had low AP scores regardless of encoding. Thus, in contrast to homopeptides, nonpolar beads associated with the E-flag appear to promote aggregation, consistent with the assumptions made in earlier works.^49,50,53,54^ This may be attributed to the heterogeneity of the side chains, which can exert a disruptive effect in the polar backbone packing similar to that of apolar beads. Notably, this effect appears to be even more pronounced when such apolar beads are present in Met-containing peptides. In fact, the E-flag often yields the highest AP values or values close to the maximum, especially for hexapeptides. Only one hexapeptide shows a higher AP under the Cluster encoding, and this occurs for the peptide that is consistently predicted to aggregate most strongly across all flags. Although PEPFOLD3 shows the highest AP in four hexapeptides and a similar AP score to E-flag in five, the differences between the two encoding groups are not statistically significant (Table S7). For decapeptides, Cluster encoding shows the highest AP in five peptides and is second-highest in the other five, with PEPFOLD3 taking first place in all of the latter. This trend suggests that, while the E-flag may still seem adequate for decapeptides, its ability to promote aggregation diminishes with increasing peptide length, making the use of more detailed ss encoding increasingly important and likely reflecting a greater role of conformation in modulating AP scores.

**Figure 5:**
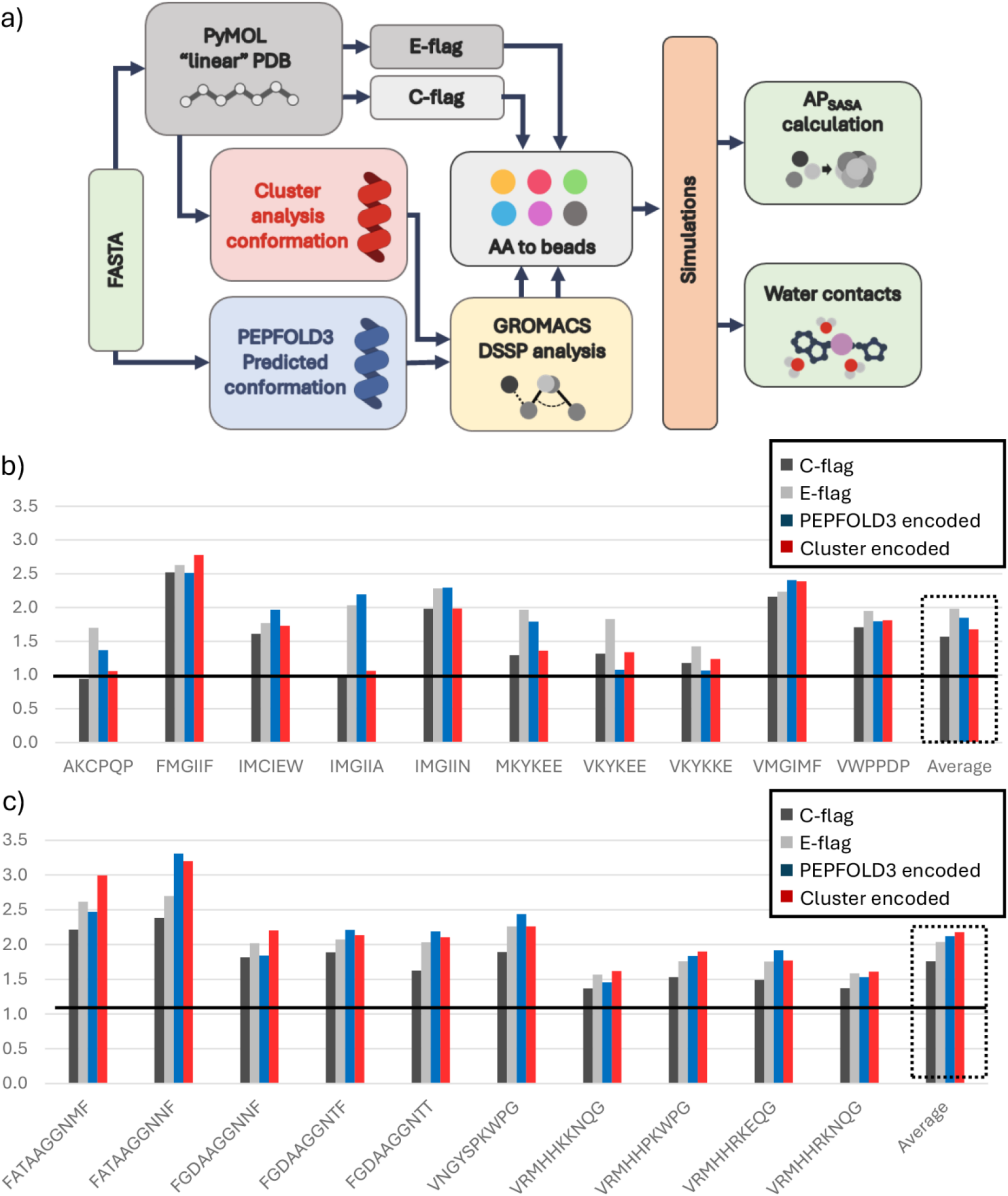
Impact of custom-encoded peptide conformation on AP_SASA_ and water contacts. (a) The following encoding was used: (i) C-flag (polar), (ii) E-flag (nonpolar), (iii) conformations from the dominant clusters from cluster analysis (Cluster) and (iv) fold predictions from PEPFOLD3. For iii and iv GROMACS DSSP analysis was performed to encode their respective conformations as ss input during simulation setup. The histograms show a set of (b) hexapeptides and (c) decapeptides simulated for 1000 ns using four types of backbone encoding: C-flag (black), E-flag (gray), PEPFOLD3 (blue), and Cluster (red). Peptides with scores close to the line (AP = 1) are considered to have no aggregation. The far right set of bars represents the average AP_SASA_ of each encoding group.

To provide an overall view of how each ss flag influences AP, we calculated the average AP score per encoding set across hexa- and decapeptides (Figure 5). SASA graphs (Figures S13 and S14) confirmed the convergence of the simulations within 1000 ns to a stable SASA plateau, and the AP values were computed over the final 50 ns lying within this flat region. For decapeptides, the differences between the E-flag, PEPFOLD3 and Cluster encodings are less pronounced than in hexapeptides (Figure 5b) and statistical tests showed that the differences are not significant (Table S7). The only significant difference was observed between the C-flag, which consistently gave lower AP scores compared to other encodings. This suggests that the choice of the ss flag may not critically impact peptide selection in screening procedures for high-AP scoring peptides. Indeed, the two best performing decapeptides (FATAAGGNNF and FATAAGGNMF) are consistent across all encodings, and the third (VNGYSPKWPG) only shifts slightly in ranking when using PEPFOLD3 encoding. Overall, the top three decapeptides are consistently identified by all methods. The same applies to the next three highest-scoring sequences (FGDAAGGNTF, FGDAAGGNTT, and FGDAAGGNNF), which all show AP > 2 values across encodings, except FGDAAGGNNF with PEPFOLD3 backbones. This consistency suggests that, for decapeptides with high AP-scores, the specific ss encoding used is unlikely to strongly affect the outcome of aggregation-based selection. In contrast, hexapeptides show much greater variability across ss encodings. While the top-performing peptide and the top three are consistent across all encodings, the fourth (IMGIIA) would be entirely missed using the Cluster or C-flags, both of which assign completely polar backbone beads. At least two other peptides show similar enhancement when nonpolar backbone beads are used. VKYKEE, for instance, aggregates more strongly under the E-flag, but not under PEPFOLD3, probably because its PEPFOLD3 backbone is mostly polar. Interestingly, IMGIIA also shows enhanced aggregation with both E-flag and PEPFOLD3 encodings, despite its PEPFOLD3 assigned backbone being largely polar. This makes it difficult to draw a clear correlation between the E-flag and PEPFOLD3 input. It is possible that differences in side chain hydrophobicity—IMGIIA being more hydrophobic than charged VKYKEE—contribute to this discrepancy, but resolving such multivariable effects would require a larger data set. However, it is evident that 4 out of the 10 hexapeptides change their position in the AP ranking depending on the ss flag used. If an AP threshold of 2 is applied, as commonly done in previous studies^49,50^ two sequences would be correctly identified using E-flag or PEPFOLD3, but missed using the Cluster or C-flags. An additional four peptides would be considered aggregating if a lower AP threshold were used,^55^ three of which show a strong dependence on the applied ss flag. These results suggest that, while the E-flag consistently yields the highest AP values for hexapeptides, it also performs reliably for decapeptides, despite not always being the top-scoring method. Its performance remains close to that of the best-performing encodings, ensuring that key aggregating sequences are not missed. However, the superior results obtained with Cluster and PEPFOLD3 encodings in specific decapeptides indicate that, as the peptide length increases, more accurate secondary structure information becomes increasingly important. Interestingly, the differences observed between fully polar Cluster-encoded sequences and those using the C-flag, which are also fully polar but differ in bonded parameters, suggest that the contribution of bonded terms may be less negligible than initially assumed, especially in longer peptides. This highlights the need to incorporate realistic ss predictions for longer sequences, where conformational diversity and backbone flexibility play a more pronounced role in modulating aggregation behavior.

### Concentration, Log*P*, and Variations in Encoding

In this study, simulations were carried out at 42 mM for hexapeptides and 25 mM for decapeptides, adapting the concentrations to compensate for the difference in volumes occupied by longer chains. In contrast, most of the literature reports employ higher concentrations, typically ranging from 140 to 400 mM (see Table S1). Our choice of lower concentrations aimed to reduce crowding, facilitate equilibration, and yield more sensitive aggregation measurements and cleaner morphologies, which can otherwise be obscured in overly dense systems.^34^ However, peptides must exceed their critical aggregation concentration (CAC) to initiate aggregation. The relationship between computational and experimental concentrations remains largely unresolved, but the few existing comparisons^34,41,70^ suggest that CG simulation concentrations should generally be much higher than their experimental counterparts. This raises the possibility that some of the simulations presented here may not have exceeded the CAC. To investigate whether switching from polar to nonpolar backbone encodings causes some peptides to completely lose aggregation capacity or simply increases their CAC, we performed additional simulations at higher concentrations (166 mM and 498 mM) by inserting 800 and 2400 peptides per simulation box, respectively.

We selected five hexapeptides (FMGIIF, AKCPQP, IMGIIA, VWPPDP and MKYKEE) to evaluate the effect of concentration using Cluster encoding in MARTINI 2.2p (Tables S9, S10 and Figure S15). Despite the increase in concentration, the SASA profiles showed comparable equilibration times across all systems (Figures S16 and S19). While FMGIIF and AKCPQP had similar AP scores across all encodings, IMGIIA, VWPPDP and MKYKEE underperformed with Cluster encoding. Interestingly, IMGIIA changed drastically between PEPFOLD3/E-flag and Cluster/C-flag encodings. Therefore, these sequences were further investigated at increasing concentrations, using the encoding with which they performed the poorest, to determine whether the shifts in behavior were related to CAC. When simulated under increasing concentration conditions, two peptides had consistent AP values at the concentrations tested, one was FMGIIF, which consistently exceeds the AP = 2 threshold, and the other VWPPDP, which remains slightly below it. Of the remaining three, IMGIIA exceeds AP = 2 under two encodings, while MKYKEE and AKCPQP do not reach this threshold under any ss flag. Such results are aligned with the aim of the generative model, which was conditioned to generate assembling (FMGIIF and IMGIIA) and nonassembling peptides (VWPPDP, MKYKEE and AKCPQP).^55^

The results (Figures 6, S15, and Table S9) showed that only IMGIIA, MKYKEE, and VWPPDP exhibited an increase in AP when the concentration was raised to 166 mM (800 peptides per box). For IMGIIA, the AP continued to increase at 498 mM (2400 peptides per box), whereas MKYKEE’s AP dropped back to a value similar to 42 mM, suggesting that the AP does not always steadily increase with concentration. Interestingly, three of the five peptides displayed reduced AP scores at 2400 peptides compared to 800, with the most pronounced drop observed in FMGIIF, which showed the highest aggregation. Visual inspection of simulation snapshots (Figure 6c) confirms that this decrease is likely due to excessive crowding at 498 mM, which leads to unrealistic morphologies that hinder proper packing and reduce the AP score. These same images also support the absence of aggregation in AKCPQP and suggest that IMGIIA, despite not exceeding the AP = 2 threshold, forms more defined aggregates in the 2400-peptide system. However, even at this highest concentration, IMGIIA fails to pass the AP threshold, indicating that while increasing concentration can enhance aggregation scores, it may not fully compensate for an unfavorable ss assignment. This is further supported by the fact that, at higher concentrations, IMGIIA reduced its overall water contacts but retained their distribution across the backbone. To further investigate these effects, hydration profiles of IMGIIA were analyzed, as previously performed for homopeptides (Figure 6b, Tables S8 and S10). The most effective water exclusion was observed for the PEPFOLD3 encoding, closely followed by the E-flag. PEPFOLD3 also showed a more detailed profile, including a notably dehydrated region at backbone position 4, an effect not clearly captured by the E-flag. Interestingly, this dehydration trend in the backbone position 4 also appears with the Cluster encoding with 2400 peptides, showing a similar behavior to PEPFOLD3 once sufficient concentration is reached. Despite this, the hydration profiles under the Cluster flag, even at 498 mM, did not reach the levels of water exclusion observed with PEPFOLD3 or the E-flag, which both corresponded to AP > 2. In summary, concentration effects on peptide aggregation strongly depend on ss encoding. Low-polarity encodings show increased AP at higher concentrations, but still fall short of the AP levels seen in more hydrophobic backbones.

**Figure 6:**
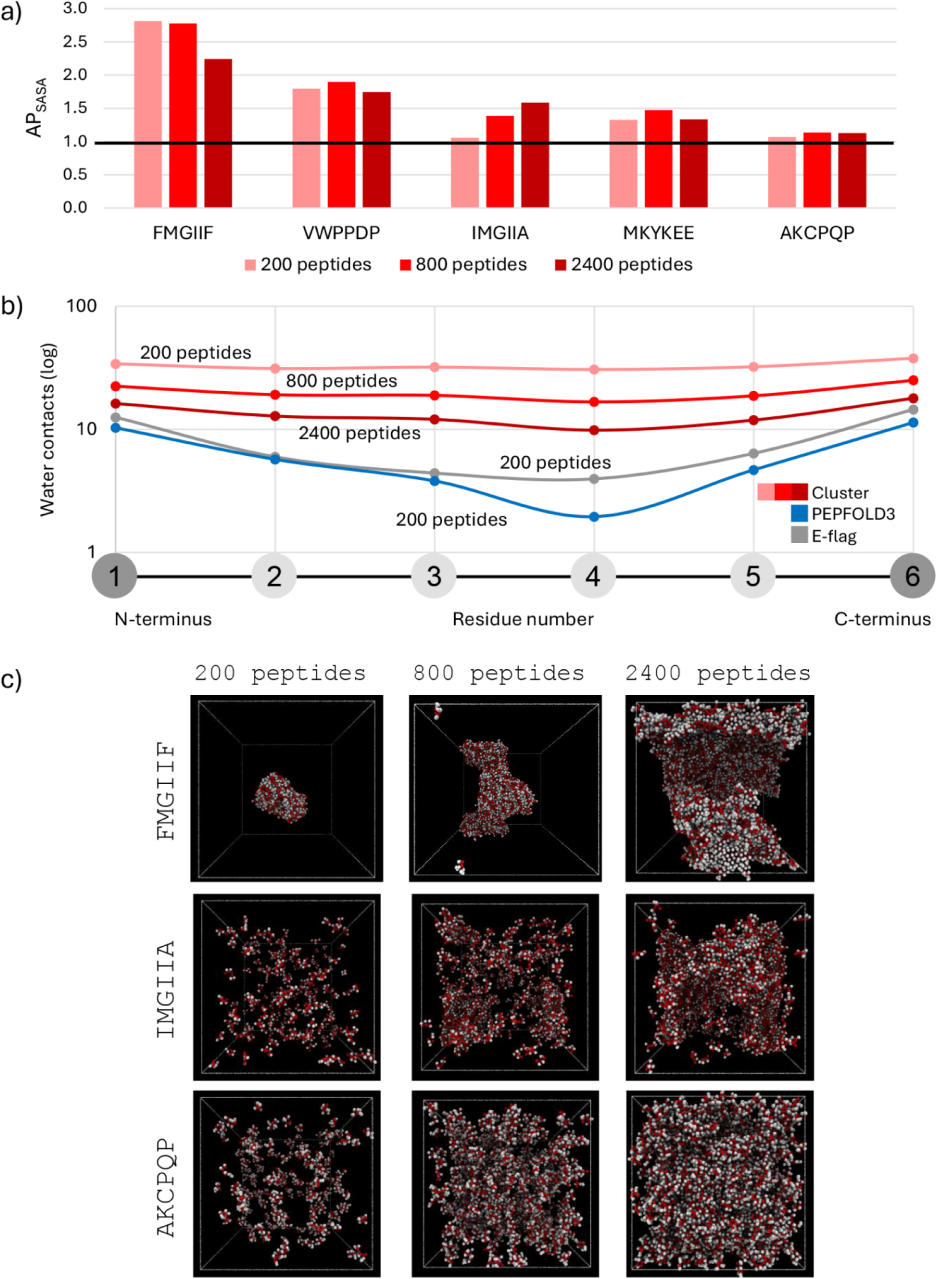
Concentration impact on aggregation in hexapeptides. (a) AP scores for selected hexapeptides with Cluster encoding showing that the AP_SASA_ of IMGIIA increased with concentration increase, while the other peptides had relatively similar or reduced scores due to crowding. (b) A water contact graph of IMGIIA comparing the radial distribution function of water molecules in different concentration scenarios (200, 800, and 2400 peptides), with E-flag and PEPFOLD3 200-peptide systems shown for comparison. While concentration increased the overall water contact numbers (Cluster), their distribution stayed similar. (c) Visualization of FMGIIF, which aggregated at all concentrations, IMGIIA, which aggregated only at higher concentrations, and AKCPQP, which did not aggregate. The red beads represent backbones and white beads are side chains.

We aimed to avoid giving the misleading impression that CG simulations are governed solely by polarity. Our initial results on homopeptides already demonstrated that this was not the case in the MARTINI model, but as we progressed to more complex sequences, this misconception became more apparent. The idea that polarity alone drives aggregation and that simply assigning the E-flag is sufficient proved increasingly inaccurate. To further investigate this, we assessed the correlations between log*P* values, as a thermodynamic measure of hydrophobicity, and AP scores obtained with different ss encodings (Figure S8). Decapeptides exhibited a higher mean *R* ^2^ value (0.651) than hexapeptides (0.217), but no encoding (C-flag) consistently produced better correlations. PEPFOLD3 yielded the most consistent *R* ^2^ values across both peptide sets, around 0.4, but was not the top performer overall, as it gave the highest *R* ^2^ values for hexapeptides and the lowest for decapeptides (Figure S8a). Ultimately, there was no strong correlation between log*P* and AP scores across the tested encodings, contradicting previous reports that found such a relationship in tripeptides.^50^ These findings support the view that estimating peptide aggregation is a complex task, especially for longer sequences, and that multiple factors beyond backbone polarity must be considered.

As mentioned above, the MARTINI 3 force field has been poorly validated for short peptides, often yielding inaccurate results for di- and tripeptides while performing better for longer sequences (≥12 amino acids).^71^ To assess its performance on the hexapeptides studied here (FMGIIF, AKCPQP, IMGIIA, VWPPDP, and MKYKEE), we repeated the simulations at the same concentrations (200, 800, and 2400 peptides per box) using MARTINI 3. In line with previous reports,^32,34,35^ MARTINI 3 failed to reproduce the aggregation behavior observed with MARTINI 2.2p, showing no aggregation in any system except FMGIIF (Figure S17 and Table S11). In particular, FMGIIF consistently shows the highest AP score across models, while other tested hexapeptides lack aggregation, with no change in behavior when increasing concentration. The water contact graphs of IMGIIA at different concentrations (200, 800, and 2400 peptides per box) with Cluster flags and 200 peptides per box with E-flag and PEPFOLD3 showed no difference in contact distribution across residues. Furthermore, the number of contacts is similar, fluctuating by only 1–2 contacts between variants and concentrations, with the exception of the most concentrated system (2400 peptides), which shows a 10-contact difference from the mean. Given that earlier work has already shown that MARTINI 3 underestimates aggregation in dipeptides, obtaining similarly negative results here suggests that this limitation also applies to hexapeptides. This result supports the notion that the reduced self-assembly propensity of MARTINI 3 decreases with peptide length. Furthermore, comparing different secondary structure encodings in MARTINI 3 (Figures S17, S18 and Tables S11, S12) revealed that using P2 backbone beads independently of secondary structure assignment largely removes the influence of secondary structure input. Interestingly, the consistent backbone polarity in MARTINI 3 reduces the variability introduced by the secondary structure encoding in 2.2p, which could make its application to short-peptide self-assembly studies more straightforward once its current limitations are addressed.

## Conclusions

Often, when new assembling peptides are proposed by generative models, the general vision in the field is that experiments are the only way to validate the new knowledge. Although this is true, CG-MD simulations have made a substantial step forward in the evaluation of peptide aggregation behavior. Our motivation was to assess whether a thorough MD evaluation of these sequences can be used as a reliable bridge between predictions of ML and experiments by studying the impact of different encodings on simulation outputs with the intention of providing a more reliable connection between computation and experiments. We showed that incorporation of near-native monomer conformations can offer a viable alternative to conventional E-flag encoding and offers a means to systematically evaluate the overall effect of secondary-structure polarity on self-assembly predictions.

Our work highlights the importance of secondary structure encoding in MARTINI CG simulations of peptide aggregation. The basic C-flag (coil) and E-flag encodings showed a strong effect on aggregation propensity (AP), with more realistic secondary structure assignments also influencing aggregation outcomes, especially for longer peptides, even favoring aggregation in some cases despite being less hydrophobic overall. Although backbone polarity plays a central role, our results show that aggregation is not governed by polarity alone. Sequence composition, peptide length, and backbone conformation all interact to shape aggregation behavior. Homopeptides and heteropeptides, for example, responded differently to the same backbone polarity, highlighting the complexity of the underlying mechanisms. In this context, MARTINI 3 ^72^ provides a practical approach to the backbone encoding problem by assigning all backbones to a polar P2 bead. However, it still fails to reproduce the self-assembly behavior of short peptides, at least up to hexapeptides. Once this limitation is addressed, the current trajectory toward developing a foldable MARTINI model capable of dynamic secondary structure adaptation would represent a major advance, enabling more accurate simulations of folding-coupled self-assembly.

Peptide concentration also influenced AP scores, particularly for more polar peptides that required higher concentrations to aggregate. It was observed that more hydrophobic secondary structure encodings reduced this need, facilitating aggregation at lower concentrations, and that overcrowding at very high concentrations could distort aggregation outcomes and their final morphology. In general, these findings highlight the need to balance concentration to avoid missing aggregation at low levels or getting misleading results from overcrowding. Overall, the E-flag performs well for short peptides and remains a practical choice for AP-based screening. However, for longer sequences (≥10), the validity of the E-flag diminishes, and more accurate secondary structure assignments, such as those from PEPFOLD3 or clustering, become necessary to reflect conformational diversity and folding effects. In this context, the use of validated tools like PEPFOLD3 becomes particularly relevant, as its secondary-structure predictions have been shown to produce more consistent and reliable self-assembly outcomes.

## Supplementary Material



## Notes

Figure S1: Final frames of 1000 ns polyglycine hexapeptide simulations; Figure S2: Final frames of 1000 ns polyglycine decapeptide simulations; Figure S3: SASA measurements of polyglycines with different backbone polarity combinations; Figure S4: Final frames of 1000 ns homopeptide simulations; Figure S5: SASA measurements of homopeptides with different backbone polarities; Figure S6: Images of cluster analysis-derived AA conformations; Figure S7: Images of PEPFOLD3 structure predictions; Figure S8: Log*P* values and AP scores of hetero-hexapeptides (42mM) and decapeptides (25mM); Figure S9: Final frames of 1000 ns CG simulations of C-flag encoded hexa- and deca-heteropeptides; Figure S10: Final frames of 1000 ns CG simulations of E-flag encoded hexa- and deca-heteropeptides; Figure S11: Final frames of 1000 ns coarse-grained simulations with encoding based on cluster analyses; Figure S12: Final frames of 1000 ns coarse-grained simulations with encoding based on PEPFOLD3 fold predictions; Figure S13: SASA measurements for hetero-hexapeptide simulations; Figure S14: SASA measurements for hetero-decapeptide simulations; Figure S15: Simulations with increasing concentrations of peptides using Cluster encoding; Figure S16: SASA measurements for heteropeptides at increasing concentrations; Figure S17: Concentration impact on aggregation in hexapeptides using MARTINI3; Figure S18: Images of the final frames of MARTINI 3 simulations; Figure S19: SASA measurements using MARTINI 3; Table S1: Summary of key publications performing CG-MD simulations of peptide self-assembly; Table S2: Polygycine homopeptide SASA scores; Table S3: Average number of water contacts per residue in polyglycine hexapeptides; Table S4: Average number of water contacts per residue in polyglycine decapeptides; Table S5: Log*P* values and AP scores of homopeptide simulations; Table S6: Detected DSSP codes using GROMACS analysis of peptide conformations obtained from cluster analysis and PEPFOLD3 structure prediction; Table S7: Statistical tests RM-ANOVA and paired difference *t*-tests for AP scores of C-flag, E-flag, Cluster encoded, and PEPFOLD3 encoded simulation groups; Table S8: Average number of water contacts per residue in hetero-hexapeptide simulations with E-flag, PEPFOLD3, and cluster encoding; Table S9: AP_SASA_ for simulations using Cluster encoding; Table S10: Average number of water contacts per residue in Cluster encoded hetero-hexapeptide simulations at concentrations of 800 (166 mM) and 2400 (498 mM) peptides per box; Table S11: AP scores for hetero-hexapeptides using Cluster encoding at three different concentrations; Table S12: Average number of water contacts per residue in hetero-hexapeptide simulations using Cluster encoding at three different concentrations (200, 800, and 2400 peptides per box) performed with the MARTINI 3 force field (PDF)
