# Supplementary material for: Secondary Structure Bead-Encoded Amphiphilicity Biases Peptide Self-Assembly Prediction in MARTINI Coarse-Grained Simulations
Source: ACS Appl Mater Interfaces. 2026 Feb 19;18(8):12353–67. doi: 10.1021/acsami.5c14754 (PMC12964340; doi:10.1021/acsami.5c14754)
Supplement: Supplementary file 1 [file am5c14754_si_001.pdf]

Supporting Information

Secondary Structure Bead-Encoded Amphiphilicity Biases Peptide Self-Assembly Prediction in MARTINI Coarse-Grained Simulations

Marko Babić,[†] Goran Mauša,[†,‡] Ivan R. Sasselli,[*,¶] and Daniela Kalafatovic[*,†,‡]

†*University of Rijeka, Faculty of Engineering, Rijeka 51000, Croatia*

‡*University of Rijeka, Center for Advanced Computing and Modelling, Rijeka 51000, Croatia*

¶*Centro de Física de Materiales (CFM-MPC), CSIC-UPV/EHU, Donostia-San Sebastián 20018, Spain*

E-mail: i.sasselli@csic.es; daniela.kalafatovic@uniri.hr

Table S1: A summary of key publications performing CG-MD simulations of peptide self-assembly. These examples show an initial screening phase performed at lower peptide concentrations and shorter production times, followed by longer simulations conducted at higher concentrations for high AP scoring sequences (as indicated in the split rows of each reference, when applicable).

Peptide length	Peptides per box	Box side size	Concentration (mmol/L)	Water type	The -ss flag	Minimization	Equilibration	Production	Force field	Ref
Tri- and pentapeptides	300 (tripeptides) and 180 (pentapeptides)	13 nm	140	W	E	Steepest descent, 10,000 steps to 200 pN energies endpoint.	NVT, 15,000 steps of 6.125 fs timestep, V-rescale temp. coupling. Production used Berendsen thermostat and barostat.	$2x10^6$ with 6.125 fs timestep (12.25 ns)	MARTINI 2.2	1
Tri- and pentapeptides	300 (tripeptides) and 180 (pentapeptides)	13 nm	230	PW	E	Steepest descent, 10,000 steps to 200 pN energies endpoint.	NVT, 15,000 steps of 6.125 fs timestep, V-rescale temp. coupling. Production used Berendsen thermostat and barostat.	$8x10^6$ with 6.125 fs timestep (49 ns)	MARTINI 2.2	1
Dipeptides	300	0.3 nm from each peptide (~10.7 nm)	400	W	E	Steepest descent, duration not mentioned.	No separate equilibration. Production run used Berendsen thermostat and barostat.	$4x10^6$ steps with 25 fs timestep (100 ns)	MARTINI 2.1	2
Dipeptides	1600	0.3 nm from each peptide (~10.7 nm)	400	W	E	Steepest descent, duration not mentioned.	No separate equilibration. Production run used Berendsen thermostat and barostat.	$40x10^6$ steps with 25 fs timestep (1000 ns)	MARTINI 2.1	2
Tripeptides	300	13 nm	230	W	E	5000 steps steepest descent, 200 pN energies endpoint.	No separate equilibration. Production run used Berendsen thermostat and barostat.	$0.5x10^6$ steps with 25 fs timestep (12.5 ns) and $4x10^6$ steps with 25 fs timestep (100 ns)	MARTINI 2.2	3
Tripeptides	300, 1200, and 2400	24 nm	30, 150, 300	PW	E	5000 steps steepest descent, 200 pN energies endpoint.	No separate equilibration. Production run used Berendsen thermostat and barostat.	$12x10^6$ steps with 25 fs timestep (300 ns) and $48x10^6$ steps with 25 fs timestep (1200 ns)	MARTINI 2.2	3
Tetra-, penta-, and hexapeptides	300	12.5 nm	255	W	E	Steepest descent, 150,000 steps of 25 fs.	No separate equilibration. Production run used V-rescale thermostat and Berendsen barostat.	$2x10^6$ steps with 25 fs timestep (50 ns)	MARTINI 2.2	4

Polyglycine simulations

Table S2: Polyglycine homopeptide SASA scores. AP_{SASA} of polyglycine hexa- and decapeptides from simulations performed at low (200 hexapeptides and 120 decapeptides) and high concentrations (800 hexapeptides and 480 decapeptides). The molar concentrations of these systems are 42 mM and 25 mM for low, and 166 mM and 100 mM for high concentrations, respectively. Backbone bead polarity is represented as nonpolar (N) or polar (P).

**Bead polarity**	**200 peptides**	**800 peptides**
NNNN	1.57	1.61
NNPP	2.04	1.97
NPNP	2.11	2.03
NPPN	1.97	2.02
PNNP	2.01	2.00
PPPP	2.06	1.98

**Bead polarity**	**120 peptides**	**480 peptides**
NNNNNNNN	1.58	1.66
NNNNPPPP	1.86	1.86
NNPPPPNN	2.13	2.16
NPNPNPNP	2.21	2.15
PPNNNNPP	2.00	2.05
PPNNPPNN	2.12	2.14
PPPPPPPP	2.20	2.11

Table S3: Average number of water contacts per residue in polyglycine hexapeptides (Gly_6), from simulations performed at concentrations of 200 and 800 peptides per box (equivalent to 42 mM and 166 mM, respectively) for different encodings. Encodings are residue 2 to 5, as terminal residues 1 and 6 are always Qd and Qa. Backbone polarity is represented by letters N for nonpolar, and P for polar beads.

Polyglycine hexapeptides' concentration: 200 peptides per box

**Bead encoding**	Res 1	Res 2	Res 3	Res 4	Res 5	Res 6
NNNN	5.2	2.2	1.9	1.9	2.2	5.2
NNPP	5.1	2.2	1.4	1.4	2.3	5.1
NPNP	4.9	2.1	1.3	1.3	2.2	4.9
NPPN	5.3	2.5	1.9	1.9	2.5	5.3
PNNP	5.1	2.4	1.3	1.4	2.4	5.1
PPPP	5.2	2.4	1.6	1.6	2.4	5.2

Polyglycine hexapeptides' concentration: 800 peptides per box

**Bead encoding**	Res 1	Res 2	Res 3	Res 4	Res 5	Res 6
NNNN	4.9	1.9	1.5	1.5	1.9	4.9
NNPP	4.9	2.1	1.3	1.3	2.2	4.9
NPNP	4.8	2.1	1.3	1.3	2.1	4.8
NPPN	4.8	2.1	1.4	1.4	2.1	4.8
PNNP	4.7	2.2	1.1	1.1	2.2	4.7
PPPP	5.3	2.6	1.8	1.8	2.6	5.3

Table S4: Average number of water contacts per residue in polyglycine decapeptides (Gly_{10}), from simulations performed at concentrations of 120 and 480 peptides per box (equivalent to 25 mM and 100 mM, respectively) for different encodings. Encodings are residue 2 to 9, as terminal residues 1 and 10 are always Qd and Qa. Backbone polarity is represented by letters N for nonpolar, and P for polar beads.

Polyglycine decapeptides' concentration: 120 peptides per box

**Bead encoding**	Res 1	Res 2	Res 3	Res 4	Res 5	Res 6	Res 7	Res 8	Res 9	Res 10
NNNNNNNN	5.4	2.5	2.0	2.0	2.0	2.0	2.0	2.0	2.5	5.4
NNNNPPPP	5.9	2.7	1.7	1.4	1.7	1.9	1.8	2.0	3.0	5.5
NNPPPPNN	5.4	2.7	1.6	1.6	1.6	1.6	1.4	1.5	2.6	5.3
PPNNNNPP	5.6	3.0	1.8	1.6	1.6	1.6	1.6	1.8	3.0	5.6
PPNNPPNN	5.5	3.1	1.9	1.5	1.4	1.3	1.3	1.5	2.7	5.5
NPNPNPNP	5.1	2.3	1.2	1.1	1.2	1.2	1.4	1.5	2.6	5.2
PPPPPPPP	5.6	3.0	1.7	1.4	1.4	1.4	1.4	1.7	2.9	5.5

Polyglycine decapeptides' concentration: 480 peptides per box

**Bead encoding**	Res 1	Res 2	Res 3	Res 4	Res 5	Res 6	Res 7	Res 8	Res 9	Res 10
NNNNNNNN	4.8	2.0	1.5	1.4	1.4	1.4	1.4	1.5	2.0	4.9
NNNNPPPP	5.3	2.3	1.3	0.9	0.9	1.0	1.1	1.5	2.7	5.0
NNPPPPNN	4.8	2.2	1.3	1.1	1.1	1.1	1.1	1.2	2.2	4.8
PPNNNNPP	5.0	2.4	1.2	1.0	0.9	0.9	1.0	1.3	2.5	5.0
PPNNPPNN	4.9	2.6	1.5	1.2	1.1	1.1	1.1	1.2	2.3	4.9
NPNPNPNP	4.8	2.3	1.3	1.0	1.0	1.0	1.0	1.3	2.5	4.9
PPPPPPPP	5.4	2.9	1.7	1.4	1.4	1.4	1.5	1.7	3.0	5.4

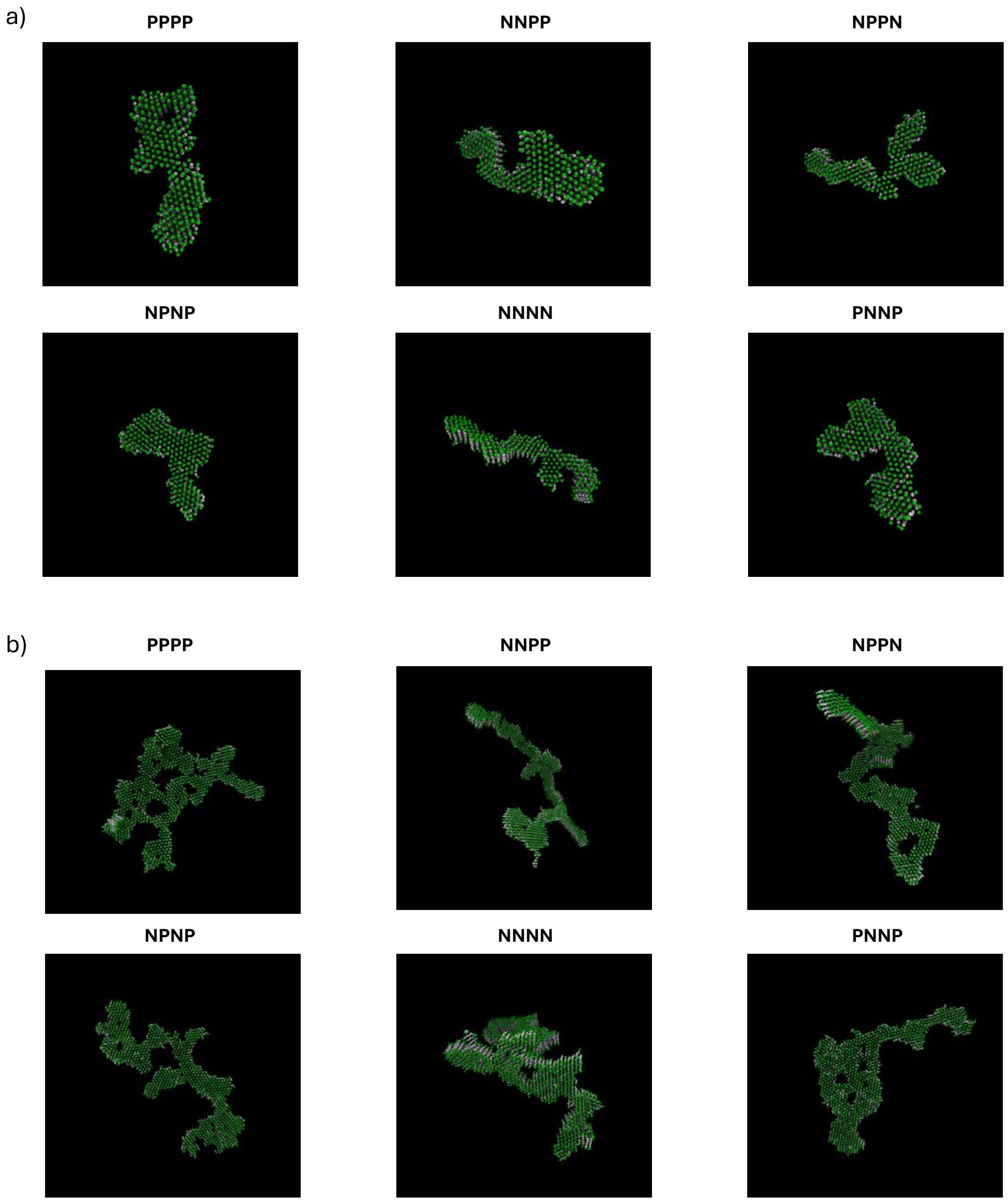

Figure S1: Final frames of 1000 ns polyglycine hexapeptide simulations. Gly_6 at (**a**) 200 peptides per box (42 mM) and (**b**) 800 peptides per box (166 mM). Charged terminal beads Qd and Qa are represented in green and beads with varying polarity are represented in white. Water and ions are removed for clarity. Backbone polarity is represented by letters N for nonpolar, and P for polar beads above each image.

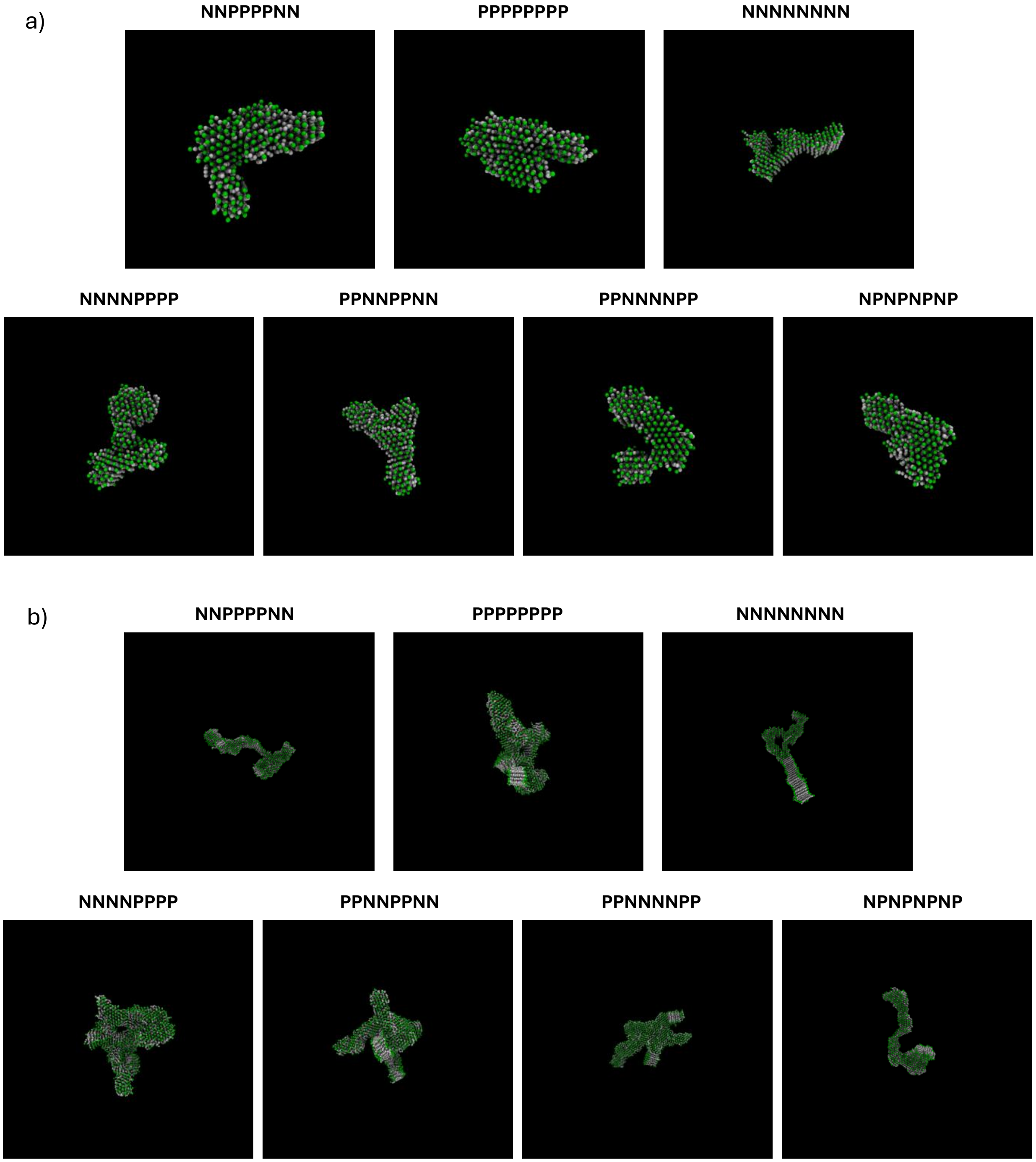

Figure S2: Final frames of 1000 ns polyglycine decapeptide simulations. Gly_{10} at (**a**) 120 peptides per box (25 mM) and (**b**) 480 peptides per box (100 mM). Charged terminal beads Qd and Qa are represented in green and beads with varying polarity are represented in white. Water and ions are removed for clarity. Backbone polarity is represented by letters N for nonpolar, and P for polar beads above each image.

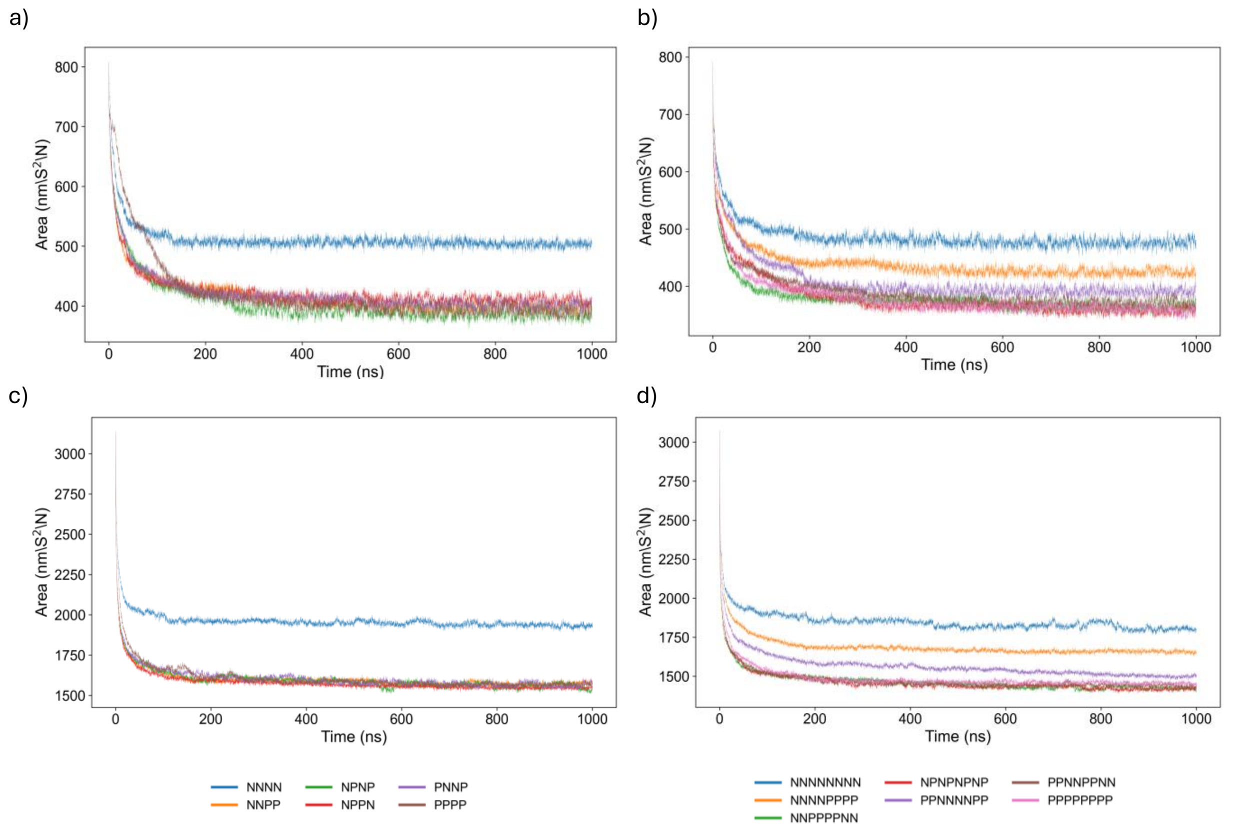

Figure S3: SASA measurements of polyglycines with different backbone polarity combinations. Hexaglycines were simulated with (**a**) 200 and (**c**) 800 peptides per box, while decaglycines were simulated with (**b**) 120 and (**d**) 480 peptides per box. Different backbone encodings used for polyglycines, either using nonpolar Nda/N0 beads (N), polar P5/P4 beads (P), or their combinations. The color-code is indicated in the legend.

Homopeptide simulations

Table S5: Log*P* values and AP scores of homopeptide simulations, accompanied by the correlation coefficient (R^2) between the log*P* and AP.

Sequence	Log*P*	Polar backbones	Nonpolar backbones
GGGGGG	-3.71	2.06	1.57
FFFFFF	6.98	3.25	3.06
IIIIII	10.81	2.22	2.28
MMMMMM	3.10	1.05	2.09
NNNNNN	-5.57	3.75	3.22
DDDDDD	-7.69	1.04	0.98
KKKKKK	-21.34	1.03	1.04
R^2	/	0.14	0.35
GGGGGGGGGG	-6.67	2.20	1.58
FFFFFFFFFF	11.03	3.33	2.69
IIIIIIIIII	17.97	2.32	2.17
MMMMMMMMMM	4.28	1.94	2.10
NNNNNNNNNN	-9.32	3.71	3.43
DDDDDDDDDD	-12.84	1.06	0.98
KKKKKKKKKK	-35.74	1.09	1.06
R^2	/	0.26	0.25

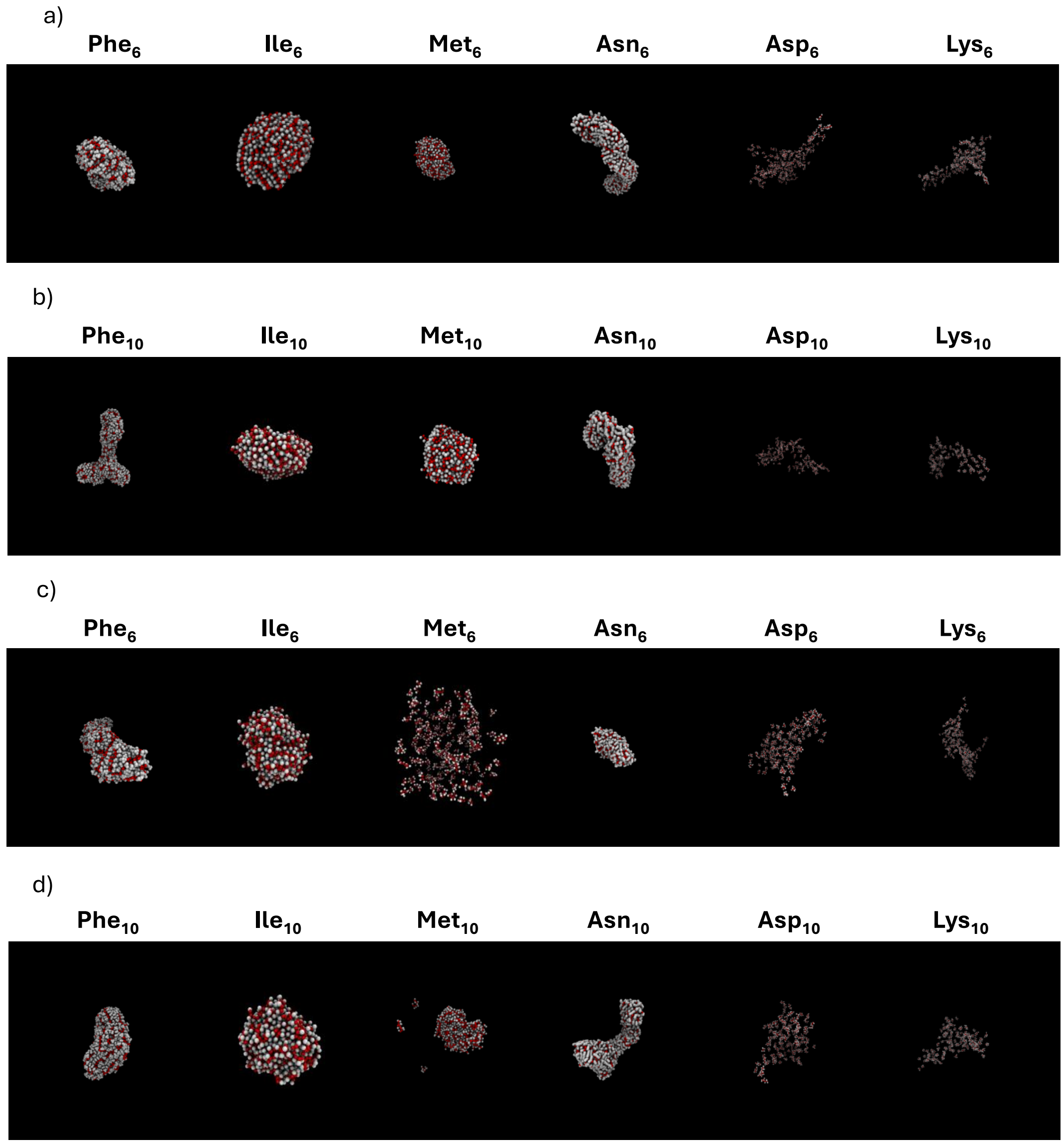

Figure S4: Final frames of 1000 ns homopeptide simulations. Snapshots of the coarse-grained simulations of homopeptides encoded with nonpolar Nda (**a** and **b**) or polar P5 beads (**c** and **d**) are shown. Panels (**a**) and (**c**) show hexa-homopeptides, while (**b**) and (**d**) show deca-homopeptides, with their corresponding sequences displayed above. Red beads represent backbones, and white beads represent side chains. Water and ions are removed for clarity. Images were clustered using GROMACS trajectory conversion, which may introduce apparent structural features in Asp and Lys homopeptides although they did not exhibit aggregation.

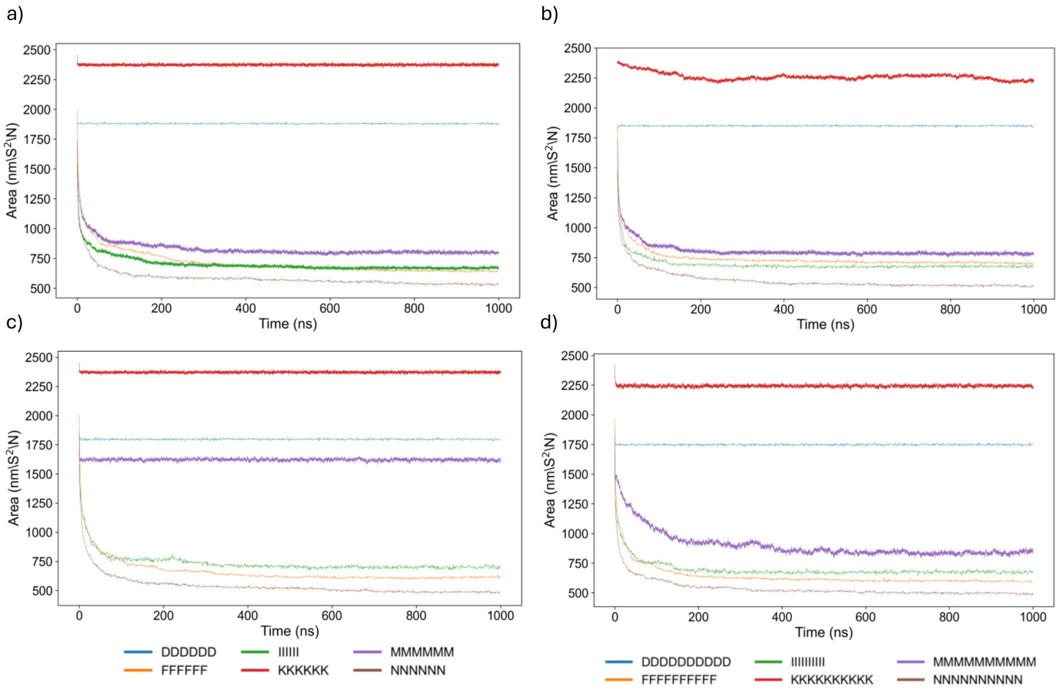

Figure S5: SASA measurements of homopeptides with different backbone polarities. Results for hexa-homopeptides simulated with (**a**) nonpolar or (**c**) polar backbones, and deca-homopeptides simulated with (**b**) nonpolar or (**d**) polar backbones. Concentrations used in the simulations were (**a**) 200, (**b**) 120, (**c**) 800, and (**d**) 480 peptides per box, respectively.

Conformational encoding simulations

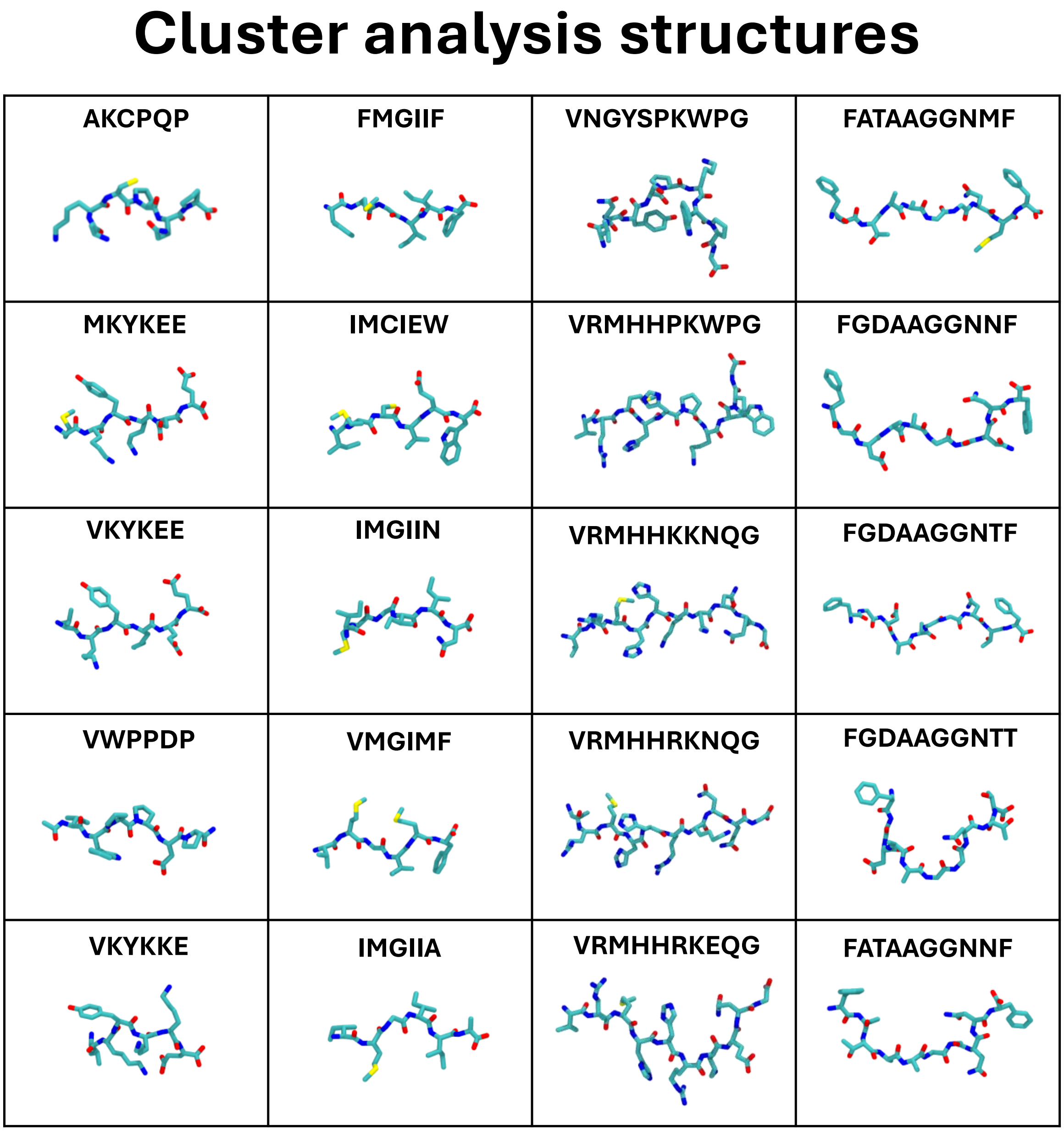

Figure S6: Images of cluster analysis-derived AA conformations. Snapshots of the representative conformation in each dominant cluster from the analysis (see Methodology).

PEPFOLD3 structures

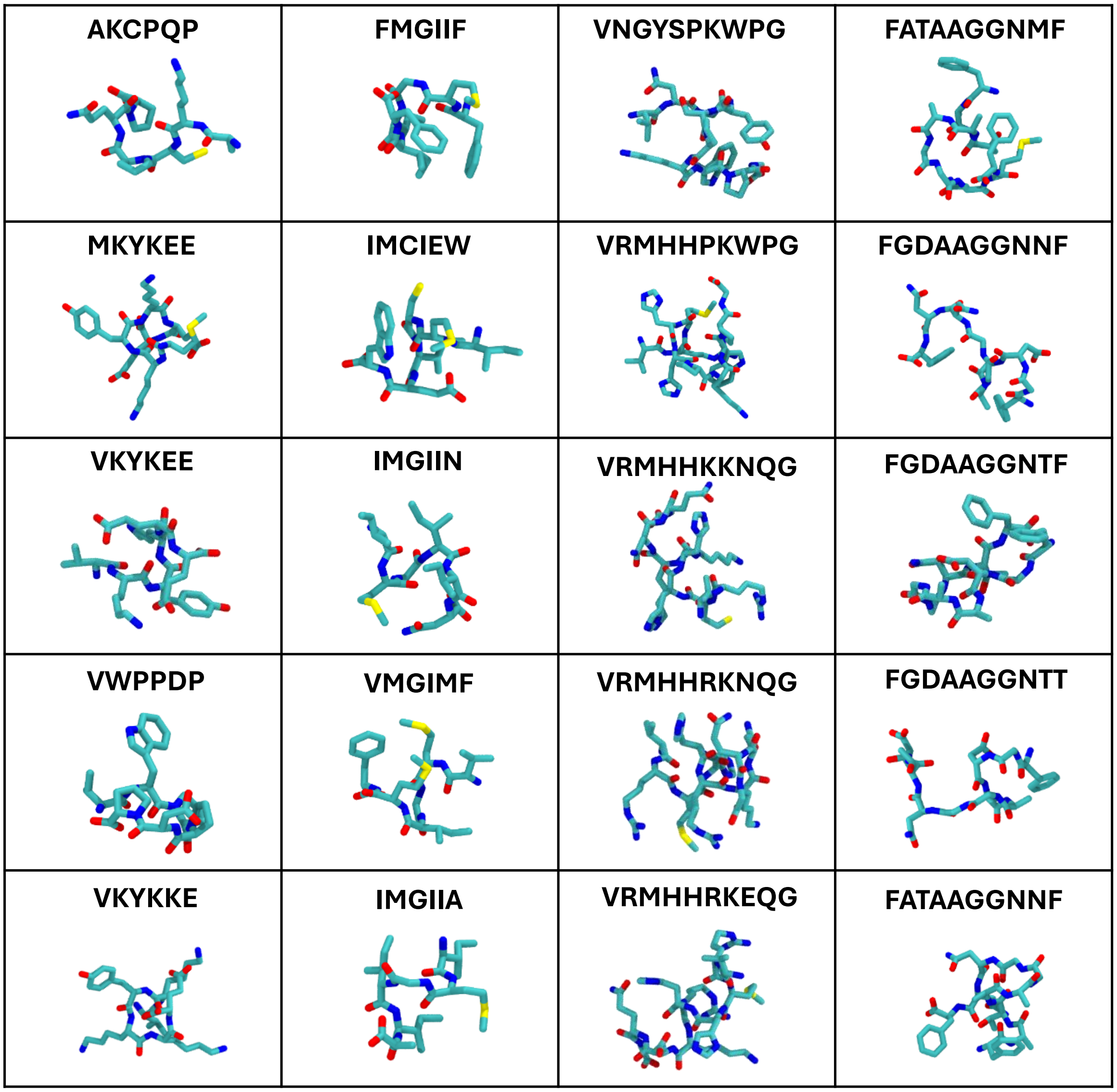

Figure S7: Images of PEPFOLD3 structure predictions. Snapshot of the lowest-energy conformation of each peptide (see Methodology). These represent the predicted lowest-energy conformations of each peptide.

Table S6: Detected DSSP codes using GROMACS analysis of peptide conformations obtained from cluster analysis and PEPFOLD3 structure prediction. Flags indicated by "~", P, S, C, B, I, G, or <no encoding> result in polar beads, while flags indicated by E, T, and H result in nonpolar beads.

Sequences	Cluster flags	PEPFOLD3 flags
IMGIIA	~~~~~~	~GGGT~
IMCIEW	~PPP~~	~HHHH~
FMGIIF	~~~~~~	~HHHH~
MKYKEE	~PPP~~	~GGG~~
VKYKEE	~~~~~~	~GGGT~
AKCPQP	~~SPP~	~~TT~~
VMGIMF	~PPP~~	~HHHH~
IMGIIN	~~~~~~	~HHHH~
VKYKKE	~~SS~~	~GGGT~
VWPPDP	~PPPP~	~~SS~~
VNGYSPKWPG	~~~PPTTS~~	~~SS~TTS~~
VRMHHKKNQG	~PPPPPP~~~	~THHHHHH~~
VRMHHPKWPG	~~~PPPSS~~	~GGGSTTS~~
VRMHHRKEQG	~PPP~~S~~~	~TTTHHHH~~
VRMHHRKNQG	~~~~~PPPP~	~HHHHHHHH~
FATAAGGNMF	~PPPP~~~~~	~TTTTTTS~~
FATAAGGNNF	~~~S~~S~~~	~~STTTS~~~
FGDAAGGNNF	~~~~~~S~~~	~TTTTSSS~~
FGDAAGGNTF	~~~S~~S~~~	~EETTTTEE~
FGDAAGGNTT	~~~S~S~~~~	~~GGGT~S~~

a)

Sequence	LogP	C-flag	E-flag	Cluster encoded	PEPFOLD3 encoded
AKCPQP	6.16	0.94	1.70	1.06	1.37
FMGIIF	4.06	2.52	2.63	2.78	2.52
IMCIEW	3.91	1.61	1.77	1.73	1.97
IMGIIA	2.13	0.97	2.03	1.06	2.19
IMGIIN	4.39	1.98	2.28	1.99	2.30
MKYKEE	-7.99	1.30	1.97	1.36	1.79
VKYKEE	-7.99	1.32	1.83	1.34	1.08
VKYKKE	-10.38	1.18	1.42	1.24	1.07
VMGIMF	4.90	2.16	2.23	2.39	2.41
VWPPDP	-5.40	1.71	1.95	1.81	1.80
R^2	/	0.13	0.26	0.16	0.44
FATAAGGNMF	-0.41	2.21	2.61	2.99	2.47
FATAAGGNNF	-1.80	2.38	2.70	3.20	3.31
FGDAAGGNNF	-2.94	1.82	2.02	2.20	1.83
FGDAAGGNTF	-2.53	1.89	2.07	2.14	2.21
FGDAAGGNTT	-4.30	1.62	2.03	2.10	2.19
VNGYSPKWPG	-3.38	1.89	2.26	2.26	2.44
VRMHHKKNQG	-11.73	1.37	1.57	1.62	1.46
VRMHHPKWPG	-4.75	1.53	1.76	1.90	1.84
VRMHHRKEQG	-12.38	1.49	1.76	1.77	1.92
VRMHHRKNQG	-11.78	1.37	1.58	1.61	1.53
R^2	/	0.69	0.69	0.64	0.48

b)

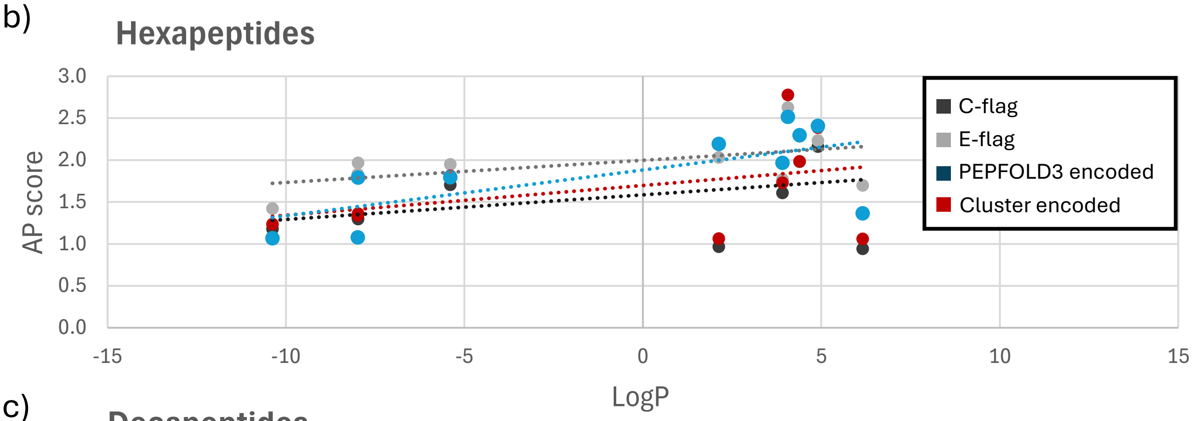

c)

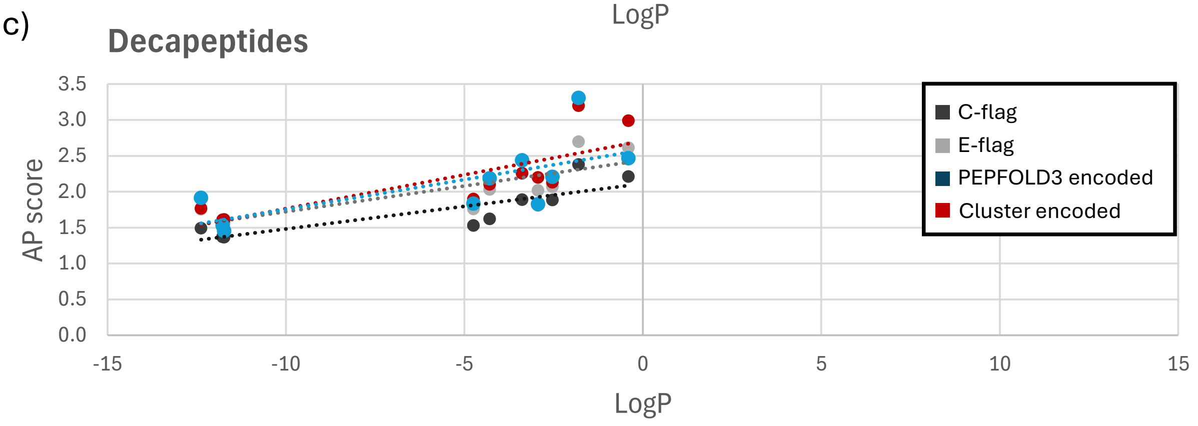

Figure S8: Log*P* values and AP scores of hetero-hexapeptides (42 mM) and decapeptides (25mM), accompanied by the correlation coefficient (R^2) between the log*P* and AP. (**a**) Log*P* values and AP scores for different encodings, accompanied by the correlation coefficient (R^2) between each encoding group and log*P*. Relationship between AP scores and log*P* for hetero- (**b**) hexapeptides and (**c**) decapeptides with added trendline for each simulation group.

Table S7: Statistical tests RM-ANOVA and paired difference t-tests for AP scores of C-flag, E-flag, Cluster encoded, and PEPFOLD3 encoded simulation groups. RM-ANOVA compared all 4 groups, while the paired t-test was used for pair-wise comparison. Although the data met the assumption of normality, Mauchly's test revealed a violation of the sphericity assumption. Consequently, the Greenhouse–Geisser correction was used to adjust the degrees of freedom. Given six t-tests were performed for deca- and hexapeptides each, the Bonferroni correction was employed. Cells colored in green indicate comparisons where the null hypothesis is rejected and the paired t-test revealed there is a statistically significant difference between two pairs of results; while the red color indicates that there was no significant difference.

RM ANOVA	Sphericity met	F-value	p-unc	p-GG-corrected	Interpretation
**Comparing all hexapeptide scores**	FALSE	5.9420	0.0030	0.0169	Statistically significant difference
**Comparing all decapeptide scores**	FALSE	15.7530	<0.0001	0.0001	Statistically significant difference

Paired t-test

HEXAPEPTIDES	Paired t-test p-values	Bonferroni corr.
PEPFOLD3 / Cluster encoded	0.2252	1.0000
E-flag / PEPFOLD3 encoded	0.1868	1.0000
Cluster / E-flag encoded	0.0268	0.1606
C-flag / PEPFOLD3 encoded	0.0587	0.3520
C-flag / E-flag encoded	0.0031	0.0186
C-flag / Cluster encoded	0.0021	0.0125

DECAPEPTIDES	Paired t-test p-values	Bonferroni correction
PEPFOLD3 / Cluster encoded	0.4345	1.0000
E-flag / PEPFOLD3 encoded	0.2941	1.0000
Cluster / E-flag encoded	0.0256	0.1535
C-flag / PEPFOLD3 encoded	0.0022	0.0134
C-flag / E-flag encoded	<0.0001	<0.0001
C-flag / Cluster encoded	0.0002	0.0009

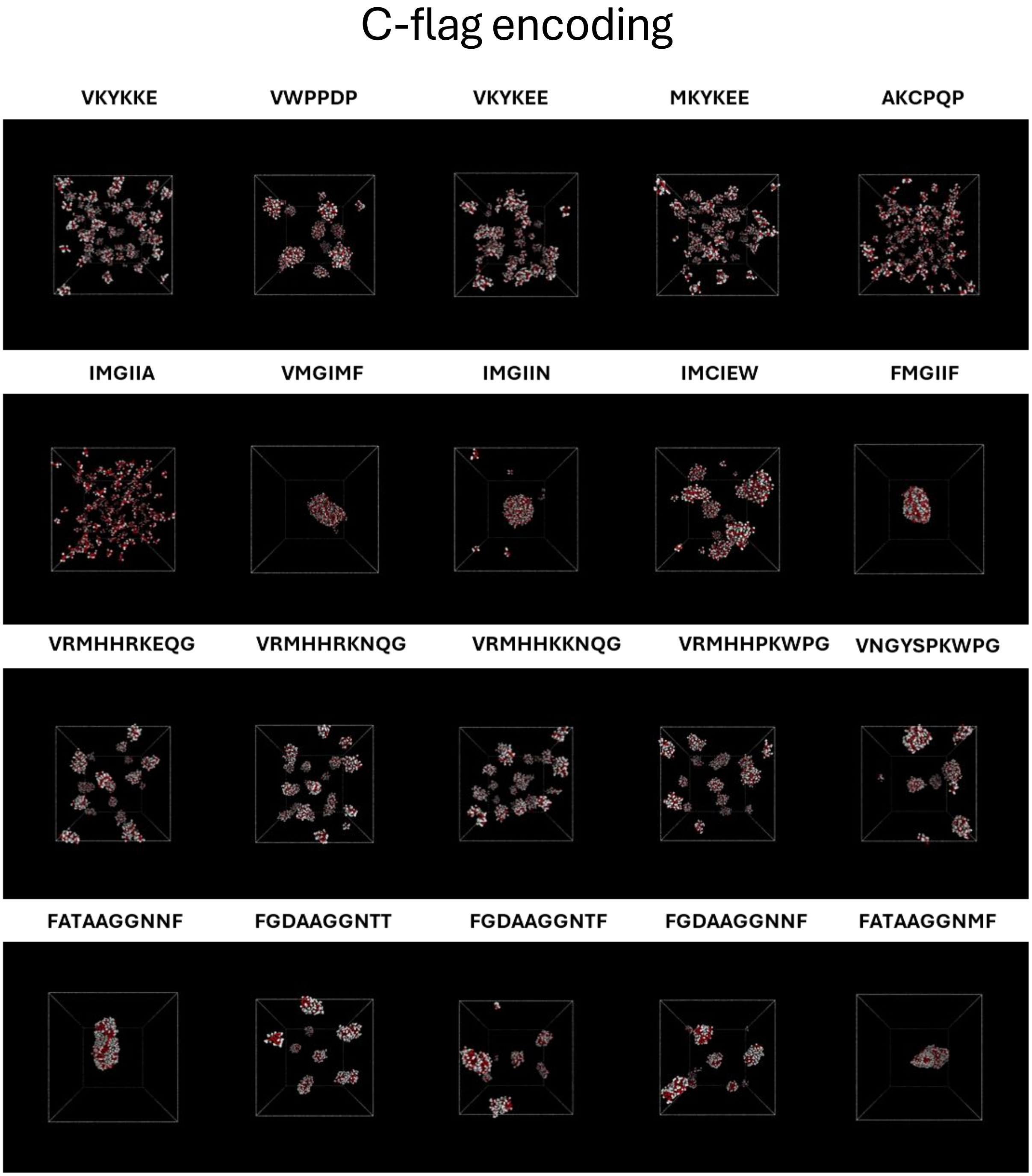

Figure S9: Final frames of 1000 ns CG simulations of C-flag encoded hexa- and deca-heteropeptides. Red beads represent backbones and white beads represent side chains. Water and ions were removed for clarity.

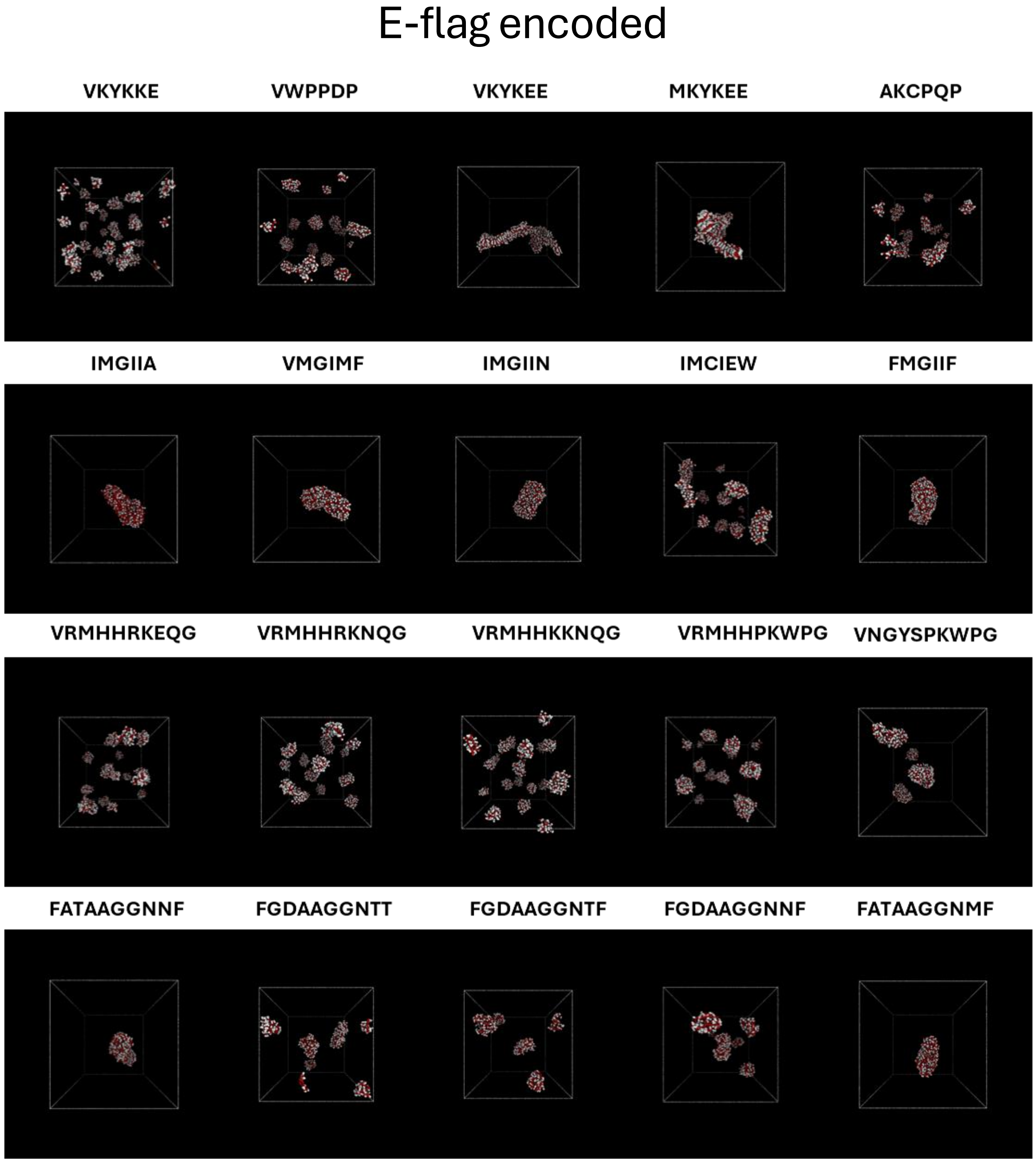

Figure S10: Final frames of 1000 ns CG simulations of E-flag encoded hexa- and deca-heteropeptides. Red beads represent backbones and white beads represent side chains. Water and ions were removed for clarity.

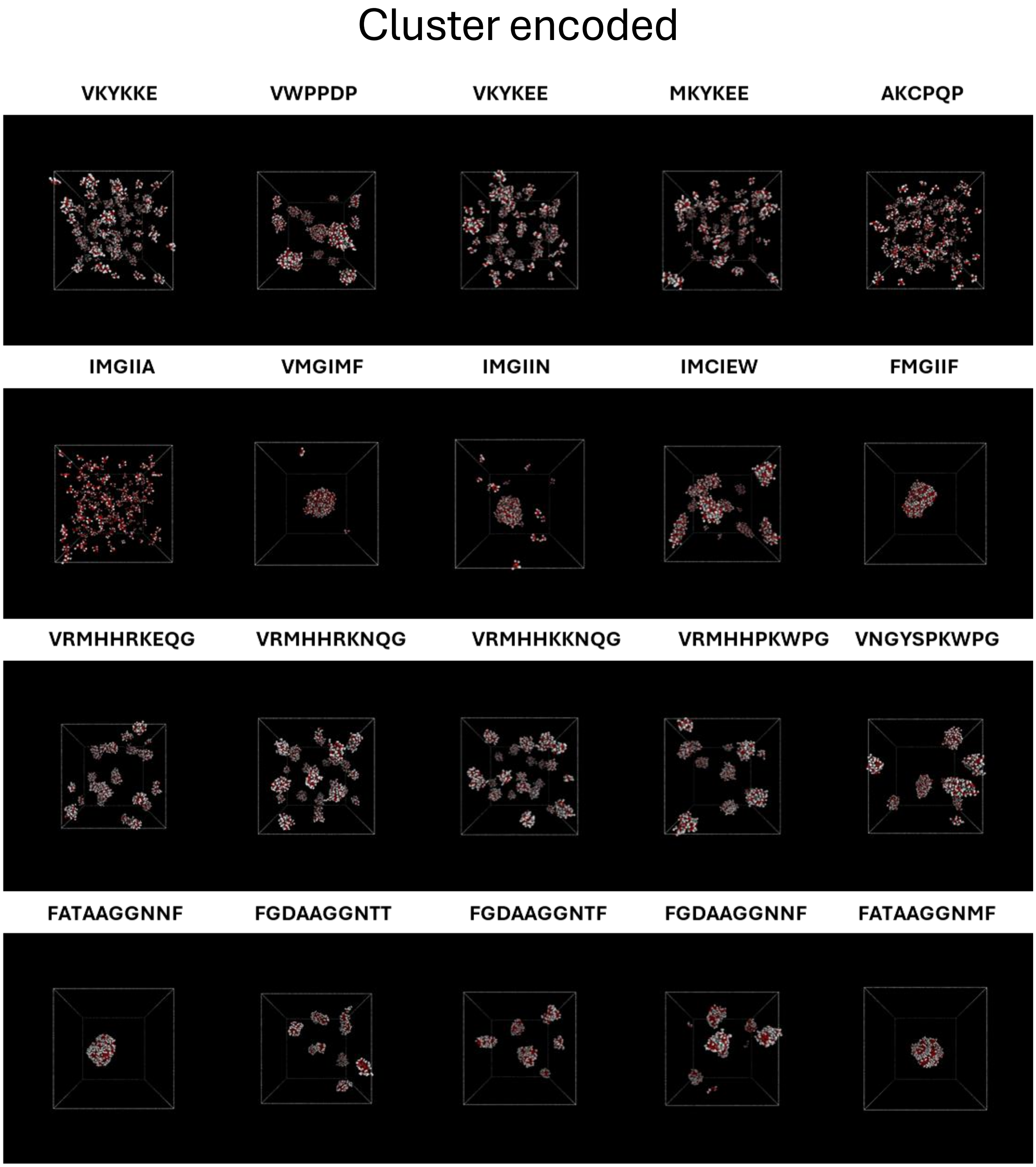

Figure S11: Final frames of 1000 ns coarse-grained simulations with encoding based on cluster analyses. Red beads represent backbones and white beads represent side chains. Water and ions are removed for clarity.

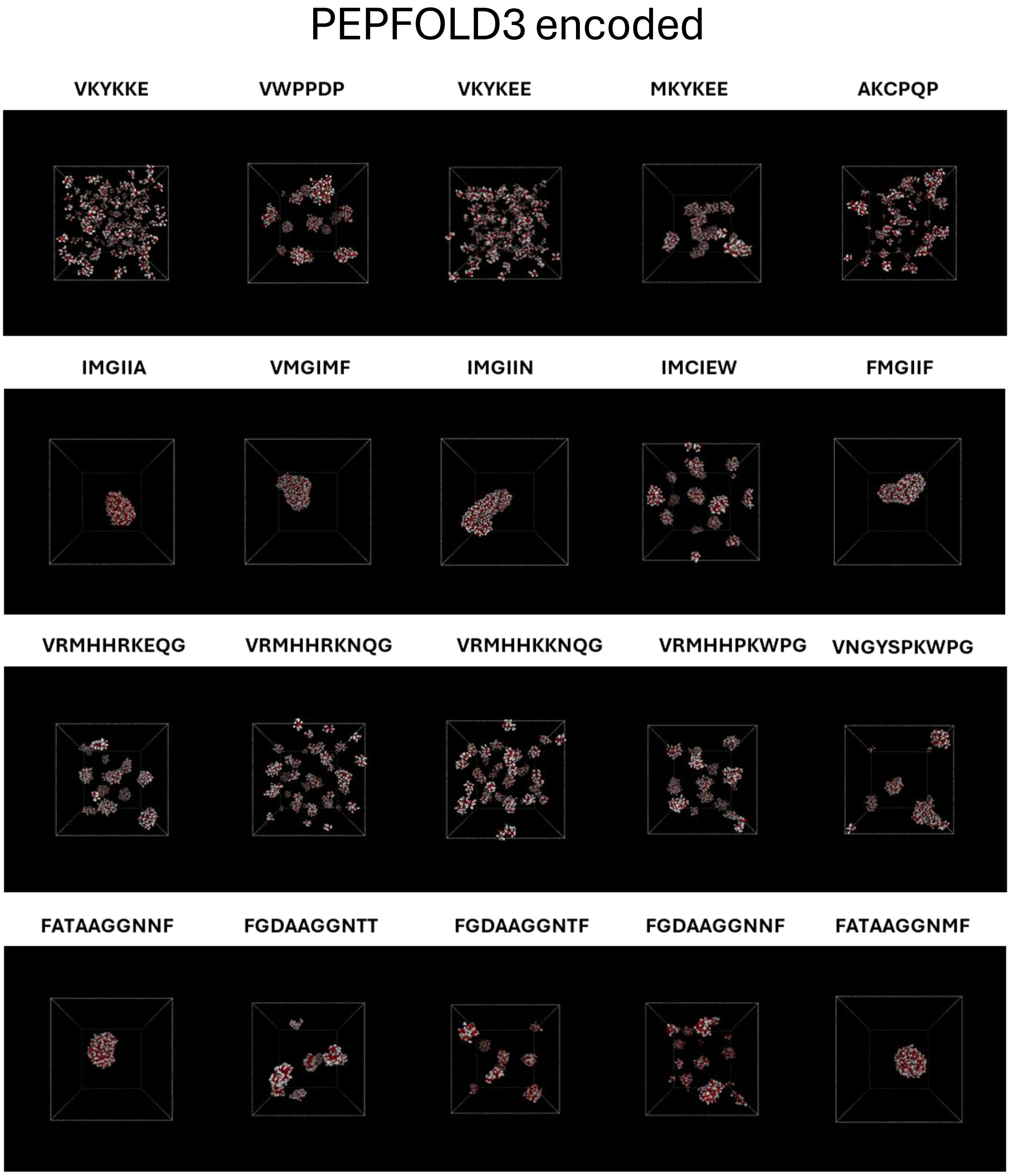

Figure S12: Final frames of 1000 ns coarse-grained simulations with encoding based on PEPFOLD3 fold predictions. Red beads represent backbones and white beads represent side chains. Water and ions are removed for clarity.

Table S8: Average number of water contacts per residue in hetero-hexapeptide simulations with E-flag, PEPFOLD3, and cluster encoding. All simulations were performed at a concentration of 200 peptides per box (42 mM).

Cluster encoded

**Sequence**	Res 1	Res 2	Res 3	Res 4	Res 5	Res 6
AKCPQP	9.0	7.2	6.5	6.5	6.7	8.0
FMGIIF	2.3	2.7	2.9	2.3	1.9	2.2
IMCIEW	4.9	3.0	2.8	3.8	5.2	4.8
IMGIIA	8.5	7.8	8.0	7.7	8.1	9.5
IMGIIN	3.7	3.1	2.9	2.5	2.7	3.5
MKYKEE	7.2	5.0	3.4	3.8	5.3	6.8
VKYKEE	7.4	4.8	3.2	3.6	5.1	6.5
VKYKKE	7.8	5.4	3.6	3.9	4.6	5.7
VMGIMF	2.6	2.5	2.6	2.2	2.0	2.1
VWPPDP	5.0	2.0	2.2	3.4	5.1	5.5

PEPFOLD3 encoded

**Sequence**	Res 1	Res 2	Res 3	Res 4	Res 5	Res 6
AKCPQP	7.5	5.3	3.4	3.1	4.5	6.2
FMGIIF	2.0	1.3	0.8	0.5	0.8	1.8
IMCIEW	3.3	1.5	1.2	2.5	3.9	4.2
IMGIIA	2.6	1.4	1.0	0.5	1.2	2.8
IMGIIN	2.9	1.4	0.8	0.4	1.1	2.7
MKYKEE	5.0	2.2	0.7	1.2	3.2	5.0
VKYKEE	8.3	6.8	6.2	6.6	6.9	7.7
VKYKKE	8.8	7.2	6.3	6.8	6.8	7.1
VMGIMF	2.3	1.0	0.5	0.4	1.0	2.0
VWPPDP	5.0	2.0	2.2	3.4	5.1	5.5

E-flag encoded

**Sequence**	Res 1	Res 2	Res 3	Res 4	Res 5	Res 6
AKCPQP	6.2	3.4	1.9	1.7	2.3	4.6
FMGIIF	2.3	1.4	1.1	0.9	1.2	2.2
IMCIEW	4.4	1.8	1.7	1.4	2.9	4.4
IMGIIA	3.1	1.5	1.1	1.0	1.6	3.6
IMGIIN	2.6	1.3	1.0	0.9	1.2	2.5
MKYKEE	5.1	2.1	0.6	0.7	2.1	4.6
VKYKEE	5.4	2.2	0.7	0.8	2.2	4.6
VKYKKE	6.9	3.7	1.8	1.9	2.5	4.6
VMGIMF	2.6	1.3	0.9	0.8	1.2	2.1
VWPPDP	4.9	1.6	2.0	1.6	3.4	5.1

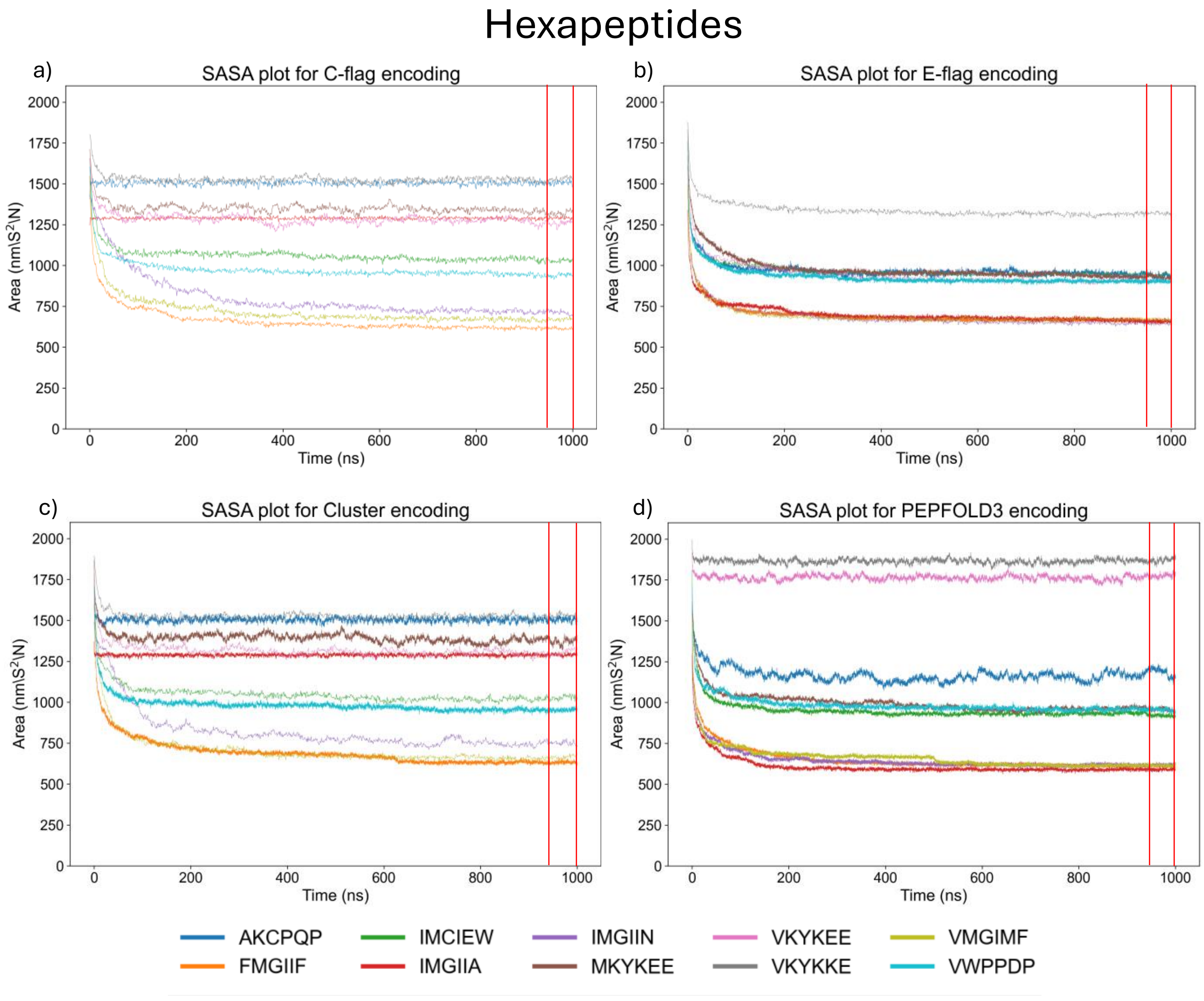

Figure S13: SASA measurements for hetero-hexapeptide simulations. The graphs show SASA throughout the 1000 ns simulation for peptides with (**a**) C-flag, (**b**) E-flag, (**c**) Cluster, and (**d**) PEPFOLD3 encoding. The vertical red lines represent the last 5% of the simulation taken to calculate AP scores (from 950 to 1000 ns).

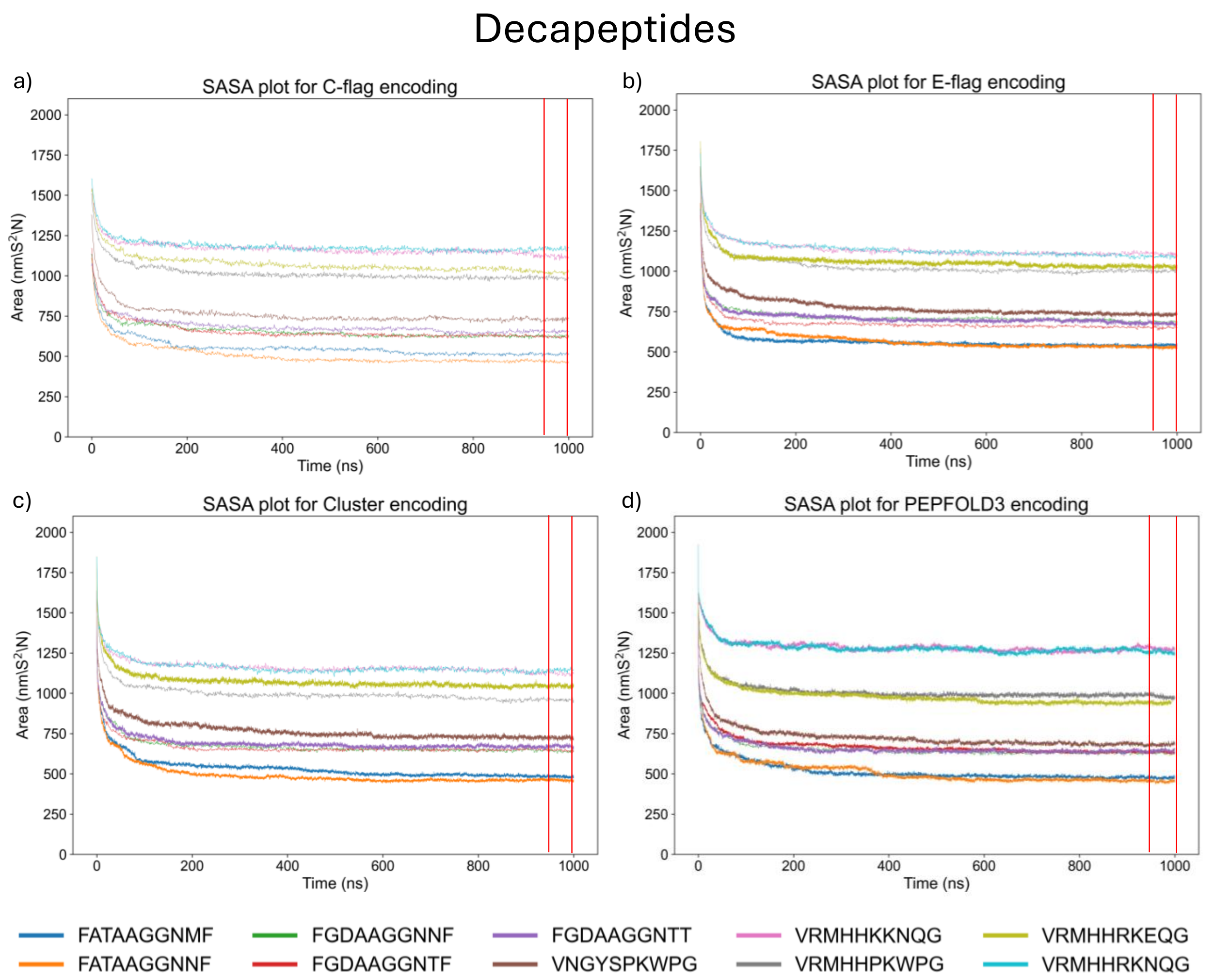

Figure S14: SASA measurements for hetero-decapeptide simulations. The graphs show changes in SASA throughout the 1000 ns simulation for peptides with (**a**) C-flag, (**b**) E-flag, (**c**) Cluster, and (**d**) PEPFOLD3 encoding. The vertical red lines represent the last 5% of the simulation taken to calculate AP scores (from 950 to 1000 ns).

Table S9: AP_{SASA} **for simulations using Cluster encoding.** The concentrations were 800 and 2400 peptides per box (166 mM and 498 mM, respectively) for hetero-hexapeptides and 480 peptides per box (100 mM) for hetero-decapeptides.

**Sequence**	AP_{SASA} **(100mM)**	AP_{SASA} **(166mM)**	AP_{SASA} **(498mM)**
FMGIIF	/	2.78	2.24
VWPPDP	/	1.90	1.74
IMGIIA	/	1.39	1.59
MKYKEE	/	1.47	1.33
AKCPQP	/	1.13	1.13
FATAAGGNNF	3.07	/	/
FATAAGGNMF	2.87	/	/
VNGYSPKWPG	2.42	/	/
FGDAAGGNTT	2.30	/	/
VRMHHRKEQG	1.77	/	/

Table S10: Average number of water contacts per residue in Cluster encoded hetero-hexapeptide simulations at concentrations of 800 (166 mM) and 2400 (498 mM) peptides per box.

800 hexapeptides per box

**Sequence**	Res 1	Res 2	Res 3	Res 4	Res 5	Res 6
FMGIIF	1.9	2.2	2.5	1.9	1.6	1.8
VWPPDP	4.3	1.5	1.6	2.8	4.5	4.7
IMGIIA	5.6	4.8	4.7	4.2	4.7	6.3
MKYKEE	6.4	4.0	2.3	2.9	4.4	5.9
AKCPQP	8.1	6.3	5.4	5.3	5.5	7.0

2400 hexapeptides per box

**Sequence**	Res 1	Res 2	Res 3	Res 4	Res 5	Res 6
FMGIIF	1.9	2.2	2.4	1.8	1.5	1.8
VWPPDP	3.9	1.3	1.3	2.0	3.5	3.5
IMGIIA	4.1	3.2	3.0	2.5	3.0	4.5
MKYKEE	5.4	3.2	1.9	2.4	3.6	4.8
AKCPQP	6.4	4.7	3.8	3.7	4.0	5.4

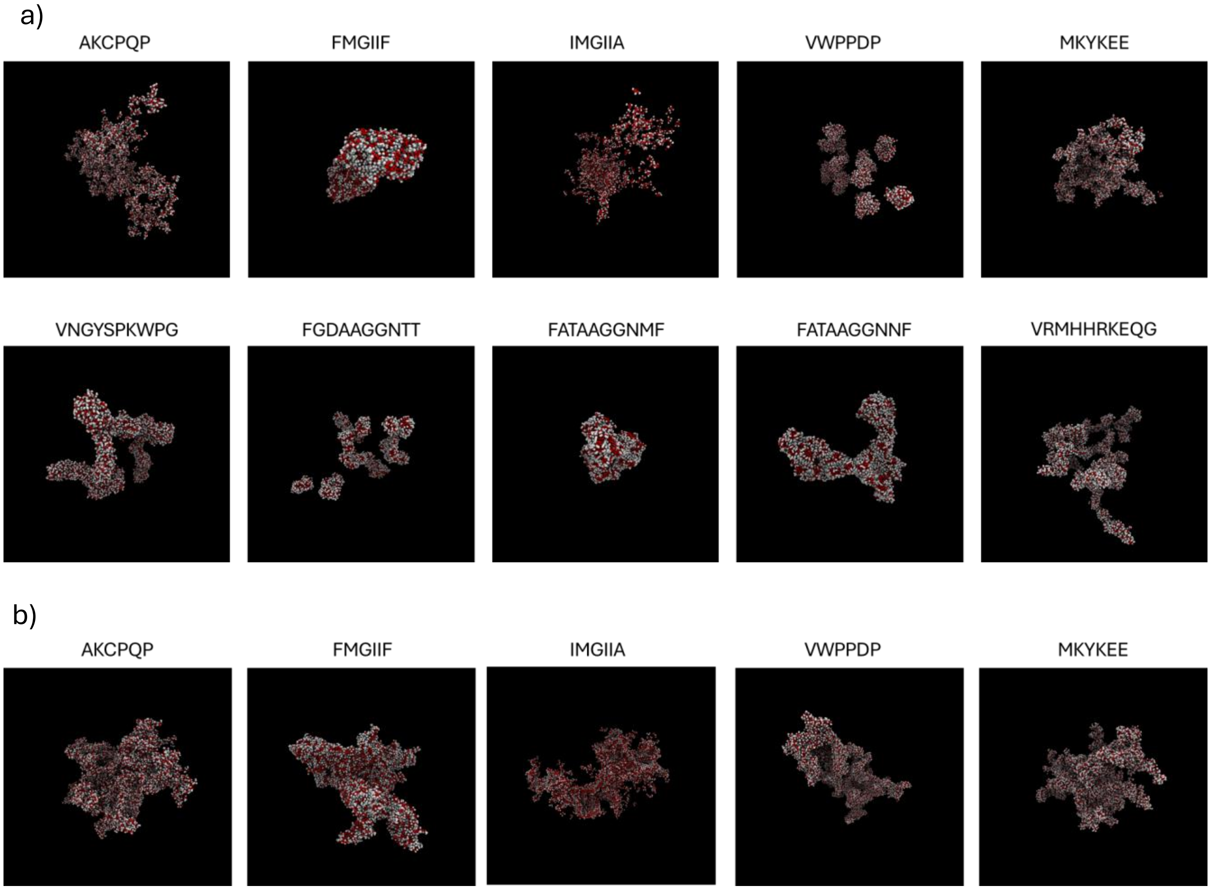

Figure S15: Simulations with increasing concentrations of peptides using Cluster encoding. Final frames of the 1000 ns simulation for selected peptides simulated using Cluster encoding with (**a**) 800 hexa- and 480 decapeptides, and (**b**) 2400 hexapeptides per box. Red beads represent backbones, white represent side chains. Water and ions are removed for clarity.

Table S11: AP scores for hetero-hexapeptides using Cluster encoding at three different concentrations (200, 800, and 2400 peptides per box equating to 42, 166, and 498 mM, respectively) simulated with the MARTINI 3 force field.

Sequence	200 peptides	800 peptides	2400 peptides
FMGIIF	1.54	2.01	1.89
VWPPDP	1.09	1.23	1.20
IMGIIA	0.96	1.09	1.05
MKYKEE	1.14	1.23	1.09
AKCPQP	1.03	1.03	1.04
IMGIIA (Eflag)	1.08	/	/
IMGIIA (PEPFOLD3)	0.96	/	/

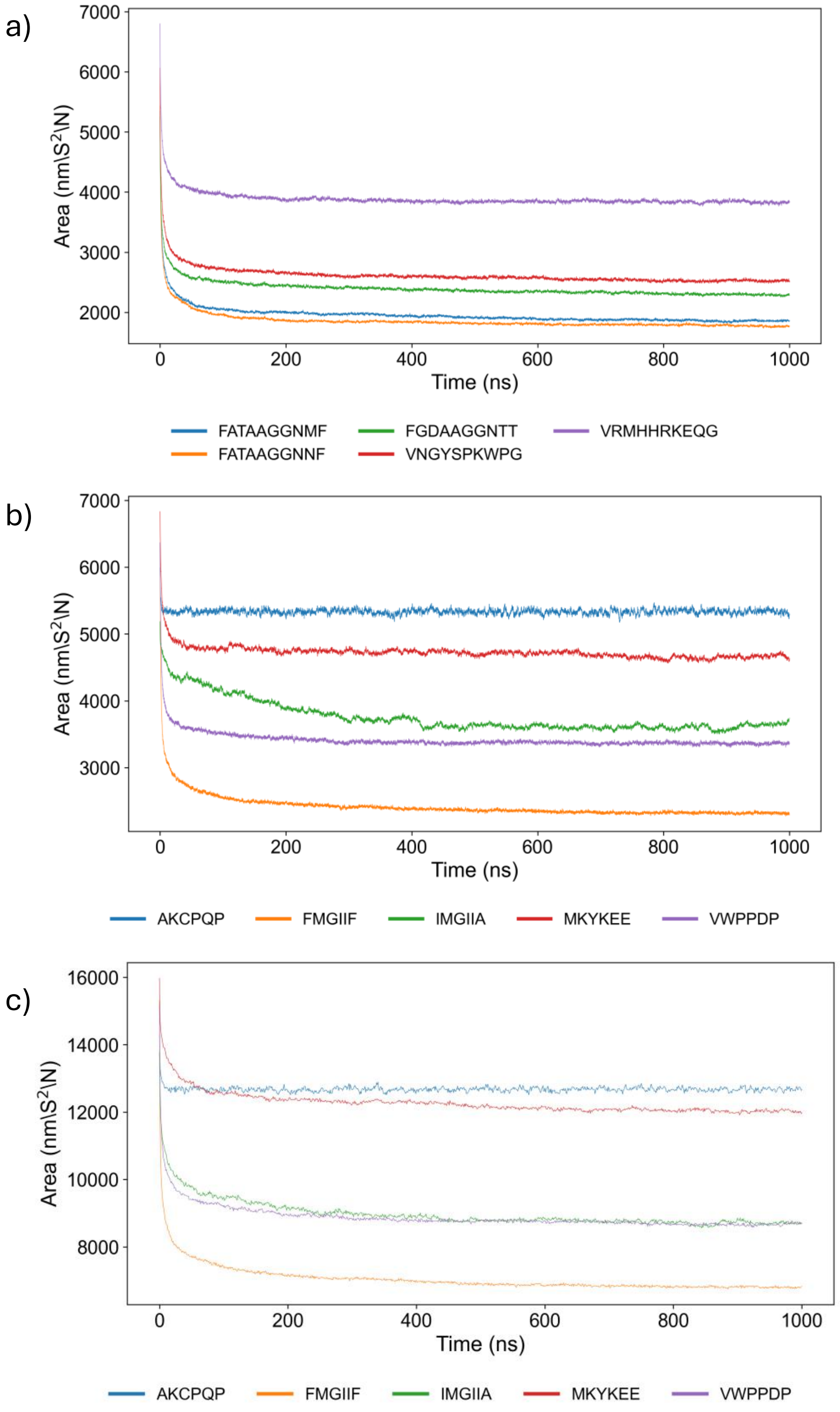

Figure S16: SASA measurements for heteropeptides at increasing concentrations. The graphs show SASA measurements throughout the 1000 ns simulation (Cluster encoding) for (**a**) 480 hetero-decapeptides, (**b**) 800 hetero-hexapeptides, and (**c**) 2400 hetero-hexapeptides per box.

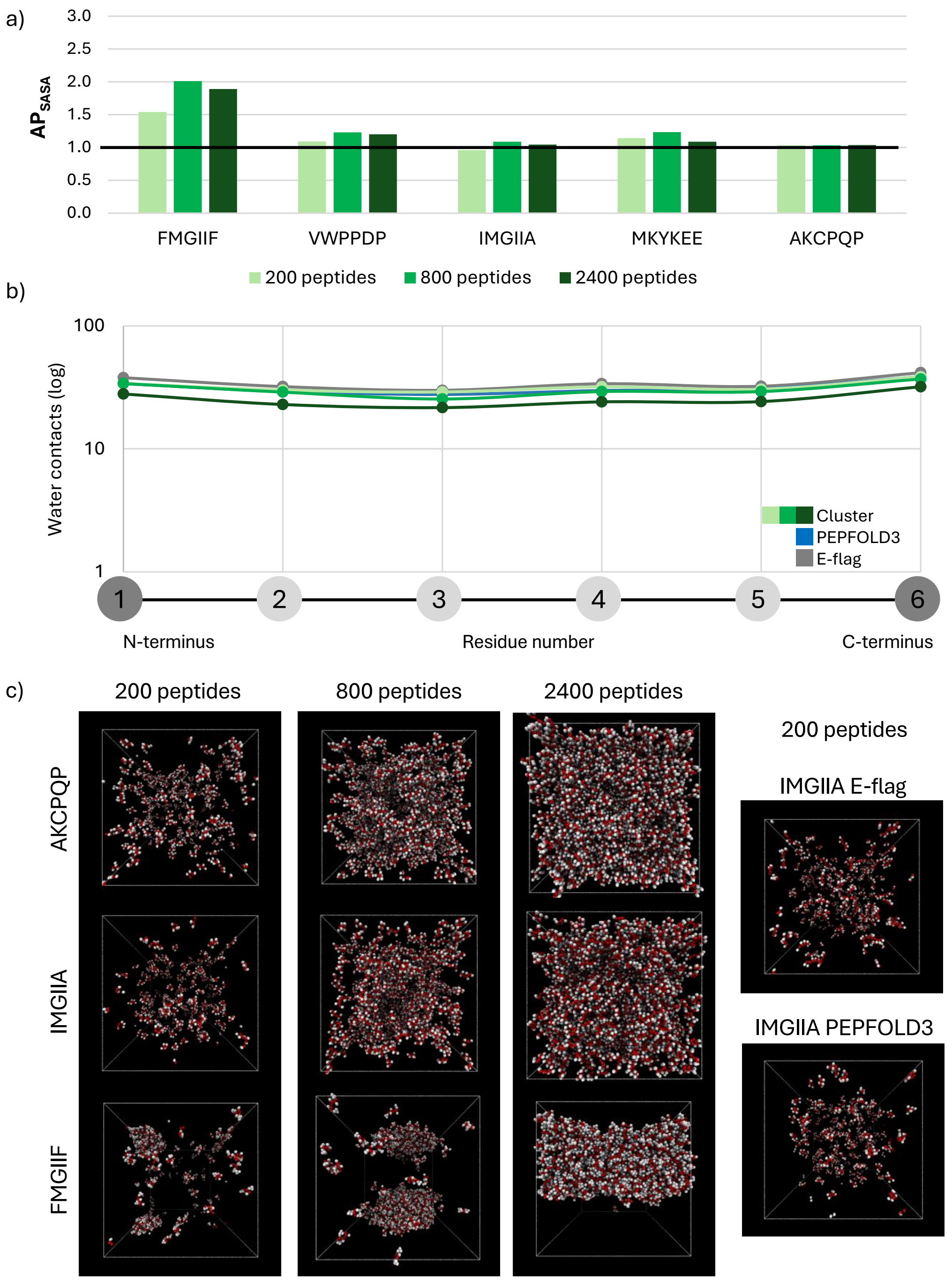

Figure S17: Concentration impact on aggregation in hexapeptides using MARTINI 3. (**a**) AP_{SASA} scores for hexapeptides with Cluster encoding. (**b**) Water contact graph of IMGIIA at different concentrations (200, 800 and 2400 peptides per box) with Cluster backbone encoding and 200 peptides per box with E-flag and PEPFOLD3. (**c**) Final frames of 1000 ns simulations. Red beads represent backbones, white represent side chains. Water and ions are removed for clarity.

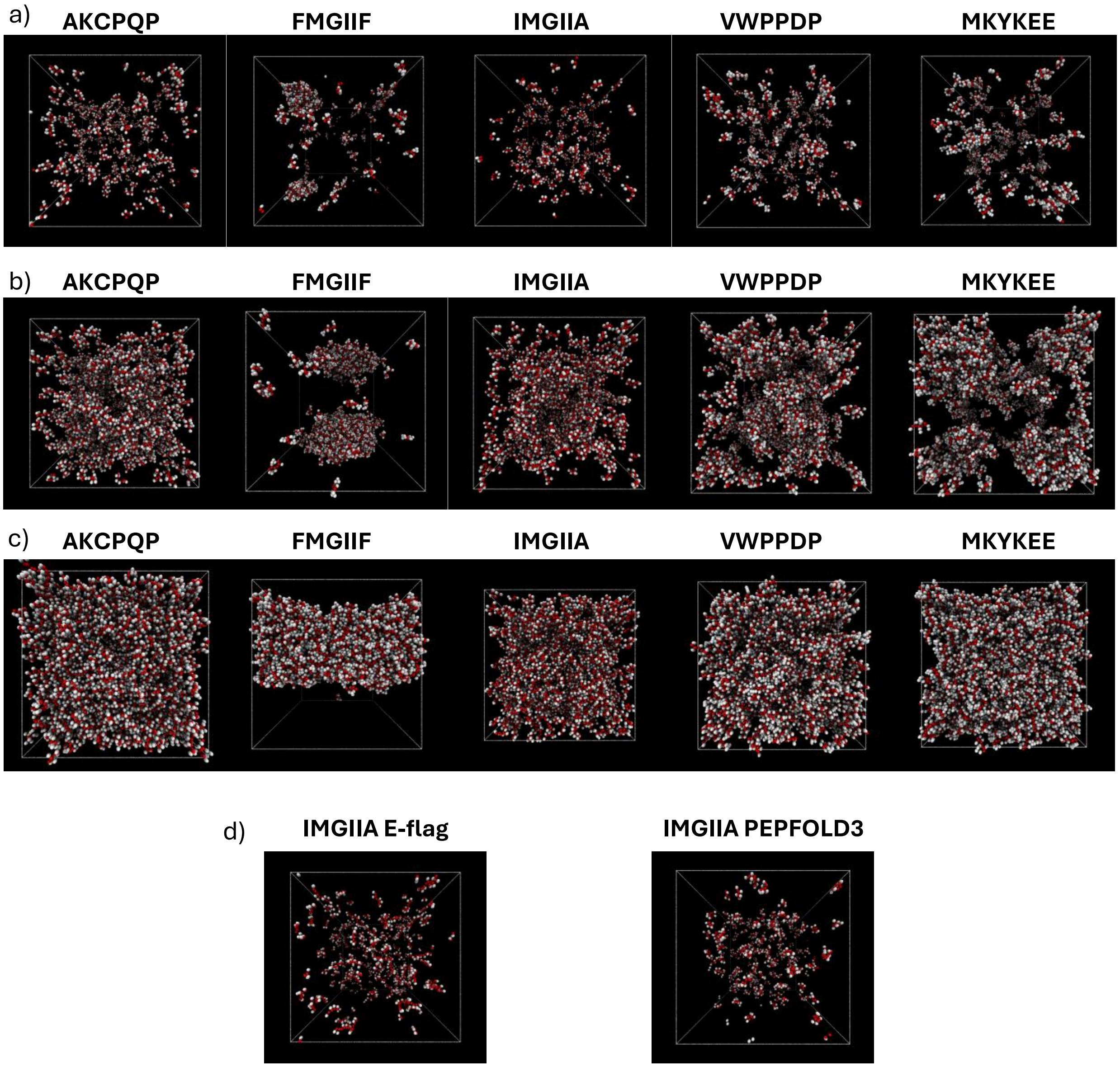

Figure S18: Images of the final frames of MARTINI 3 simulations. Selected hexapeptides were simulated for 1000 ns suing MARTINI 3 force field at 3 different concentrations (**a**) 200, (**b**) 800, and (**c**) 2400 peptides per box with Cluster backbone encoding, and (**d**) the peptide IMGIIA was additionally simulated with the E-flag and PEPFOLD3 flags using 200 peptides per box. The red beads represent backbones and white beads represent side chains. Solvent and ions are removed for clarity.

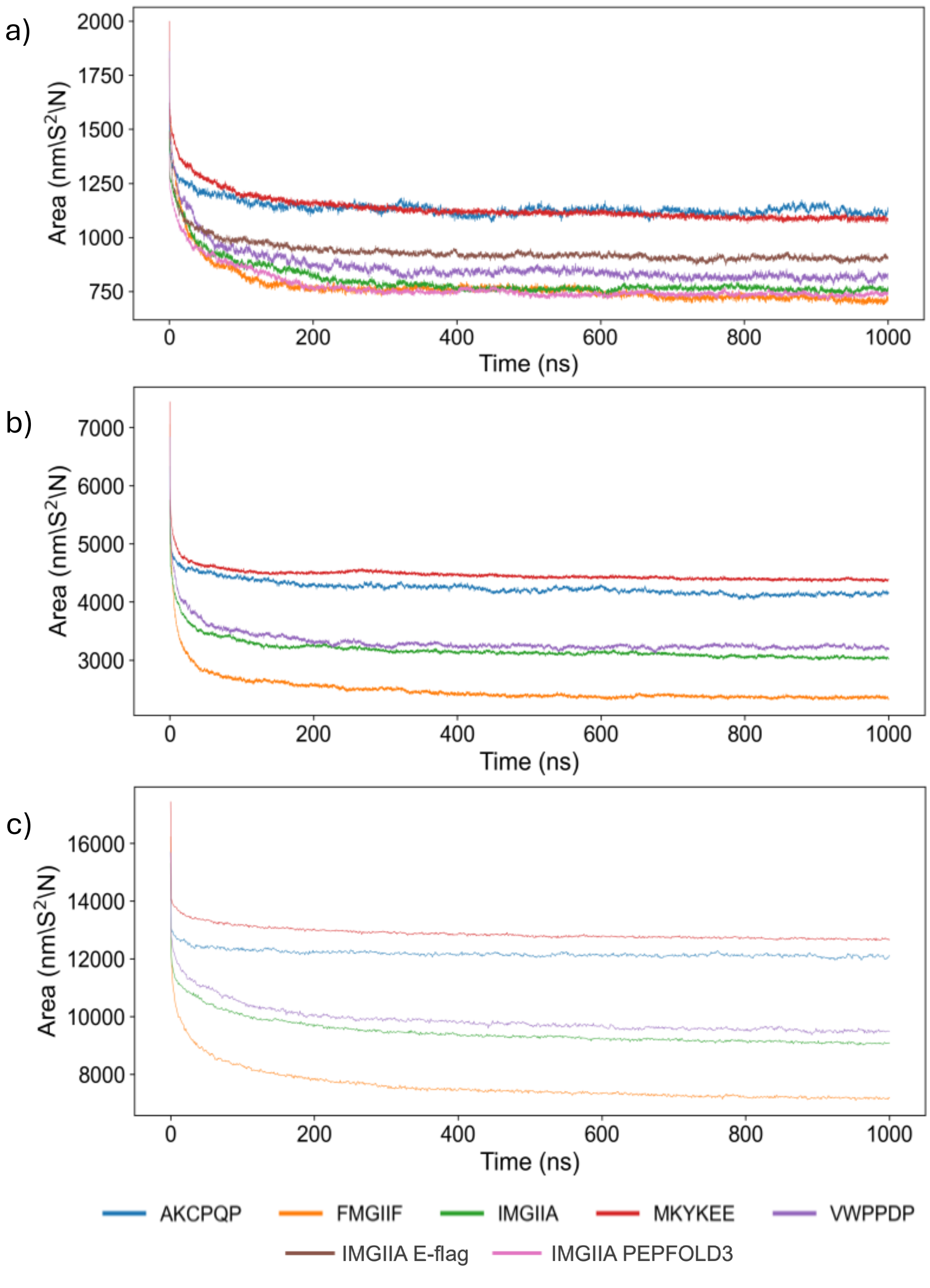

Figure S19: SASA measurements using MARTINI 3. SASA measurements for concentrations of (**a**) 200, (**b**) 800, and (**c**) 2400 peptides per box using Cluster backbone encoding. (**a**) The peptide IMGIIA was additionally simulated with the E-flag and PEPFOLD3 flags using 200 peptides per box.

Table S12: Average number of water contacts per residue in hetero-hexapeptide simulations using Cluster encoding at three different concentrations (200, 800, and 2400 peptides per box) performed with the MARTINI 3 force field.

200 hexapeptides per box

Sequence	Res 1	Res 2	Res 3	Res 4	Res 5	Res 6
FMGIIF	6.0	5.0	4.2	4.6	4.8	6.0
VWPPDP	8.4	6.9	6.4	6.4	7.1	8.8
IMGIIA	8.5	7.5	7.3	8.0	7.7	9.7
MKYKEE	6.8	6.0	5.8	5.9	5.5	6.7
AKCPQP	9.7	7.7	7.5	6.9	7.1	8.7
IMGIIA (PEPFOLD3)	8.5	7.3	6.9	7.5	7.7	9.7
IMGIIA (Eflag)	9.5	8.0	7.5	8.5	8.1	10.4

800 hexapeptides per box

Sequence	Res 1	Res 2	Res 3	Res 4	Res 5	Res 6
FMGIIF	4.5	3.5	2.9	3.2	3.5	4.3
VWPPDP	7.4	5.5	5.2	5.4	6.5	8.3
IMGIIA	8.5	7.2	6.4	7.3	7.3	9.3
MKYKEE	6.1	5.5	5.1	5.3	5.1	6.4
AKCPQP	9.4	7.4	7.1	6.5	6.7	8.3

2400 hexapeptides per box

Sequence	Res 1	Res 2	Res 3	Res 4	Res 5	Res 6
FMGIIF	3.9	3.1	2.5	2.8	3.0	3.8
VWPPDP	6.6	4.5	4.2	4.6	5.9	7.7
IMGIIA	7.0	5.7	5.4	6.0	6.1	8.0
MKYKEE	5.6	5.1	4.7	4.9	4.8	6.2
AKCPQP	8.3	6.5	6.2	5.6	5.8	7.2

References

(1) Batra, R.; Loeffler, T. D.; Chan, H.; Srinivasan, S.; Cui, H.; Korendovych, I. V.; Nanda, V.; Palmer, L. C.; Solomon, L. A.; Fry, H. C.; Sankaranarayanan, S. K. R. S. Machine learning overcomes human bias in the discovery of self-assembling peptides. *Nat. Chem.* **2022**, *14*, 1427–1435.

(2) Frederix, P. W. J. M.; Ulijn, R. V.; Hunt, N. T.; Tuttle, T. Virtual Screening for Dipeptide Aggregation: Toward Predictive Tools for Peptide Self-Assembly. *J. Phys. Chem. Lett* **2011**, *2*, 2380–2384.

(3) Frederix, P. W. J. M.; Scott, G. G.; Abul-Haija, Y. M.; Kalafatovic, D.; Pappas, C. G.; Javid, N.; Hunt, N. T.; Ulijn, R. V.; Tuttle, T. Exploring the sequence space for (tri-)peptide self-assembly to design and discover new hydrogels. *Nat. Chem.* **2015**, *7*, 30–37.

(4) van Teijlingen, A.; Tuttle, T. Beyond Tripeptides Two-Step Active Machine Learning for Very Large Data sets. *J. Chem. Theory Comput.* **2021**, *17*, 3221–3232.
